# Supplementary material for: Activating mutations in BRAF disrupt the hypothalamo-pituitary axis leading to hypopituitarism in mice and humans
Source: Nat Commun. 2021 Apr 1;12:2028. doi: 10.1038/s41467-021-21712-4 (PMC8016902; doi:10.1038/s41467-021-21712-4)
Supplement: Supplementary file 1 — Supplementary Information [file 41467_2021_21712_MOESM1_ESM.pdf]

BRAF mutations disrupt the hypothalamo-pituitary axis leading to hypopituitarism in mouse and humans

Angelica Gualtieri, et al.

Supplementary Figures.

Supplementary Tables.

Uncropped unedited Gels.

Supplementary Figure 1

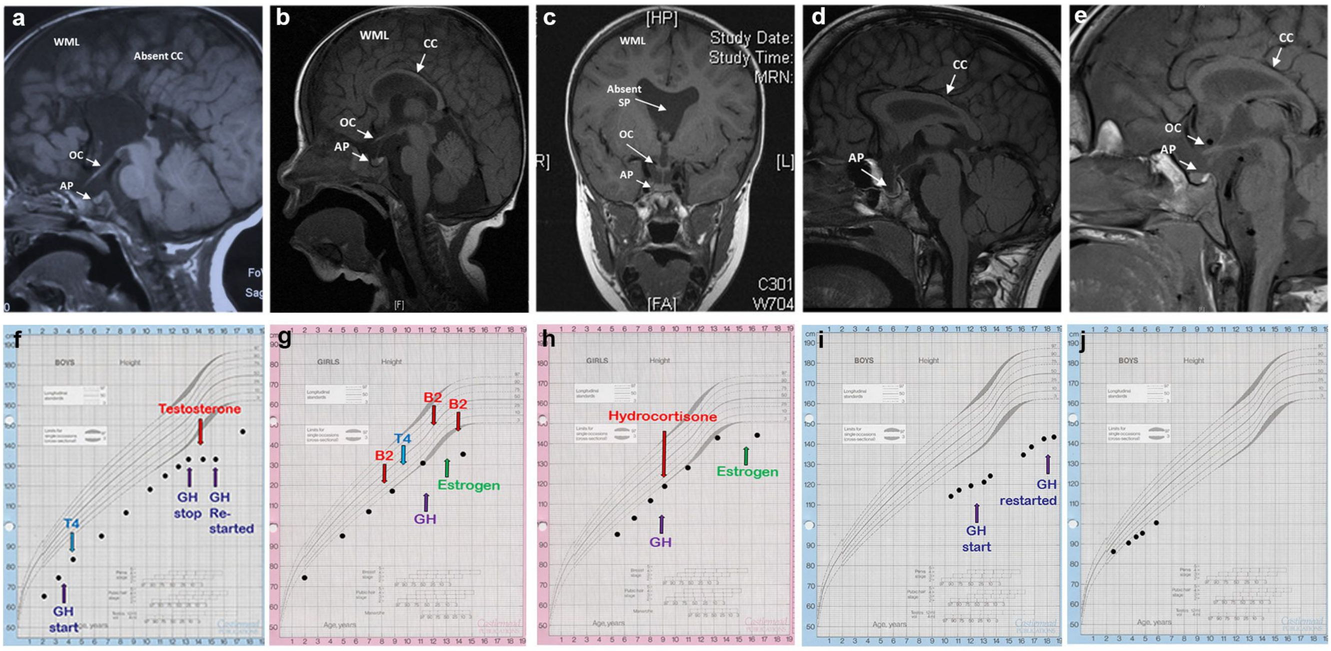

Supplementary Figure 1: a-e) MRI scans of Patients 1-5. a) Patient 1 has a small anterior pituitary and infundibulum, agenesis of the corpus callosum, an absent septum pellucidum, optic nerve hypoplasia and reduced white matter. **b)** Patient 2 has a normal pituitary, a hypoplastic corpus callosum, optic nerve hypoplasia, reduced white matter and enlarged ventricles. **c)** Patient 3 has a normal pituitary, an absent septum pellucidum, optic nerve hypoplasia, slender corpus callosum and reduced white matter. **d)** Patient 4 has an enlarged pituitary fossa. The small anterior pituitary gland was displaced to the left hand side of the fossa and there was dilatation of the infundibular recess of the third ventricle with tethering of the infundibulum. **e)** Patient 5 has a normal pituitary, hypoplasia of the corpus callosum and global underdevelopment of the brain with reduced white matter bulk. **f-j)** Growth charts of Patients 1-5. **f)** GH treatment was commenced at 3.6y in Patient 1. A six month trial of GH resulted in growth arrest. GH was subsequently restarted at 14.4y. Levothyroxine was commenced at 4.1y and testosterone treatment commenced at 14.1y. **g)** Levothyroxine was commenced in patient 2 at 9.7y and GH added at 11.4y, followed by transdermal estrogen at 13y commenced for pubertal arrest. **h)** Patient 3 was commenced on GH for short stature at the age of 7.7y. At the age of 9y, she was commenced on hydrocortisone. At the age of 15.4y, pubertal arrest necessitated the use of estrogen. **i)** Patient 4 was diagnosed with GHD at age 12.5y, and was commenced on GH treatment. He was lost to follow-up at age 13.8 years and GH was stopped at age 16y. Retesting confirmed a diagnosis of GHD. **j)** Patient 5 presented with short stature; endocrine testing at age 3.7y revealed a normal GH response to provocation. His growth rate remains suboptimal and further investigations are planned. Abbreviations: AP, anterior pituitary; CC, corpus callosum; SP, septum pellucidum; OC, optic chiasm; WML, white matter loss, T4, thyroxine; GH, growth hormone.

Supplementary Figure 2

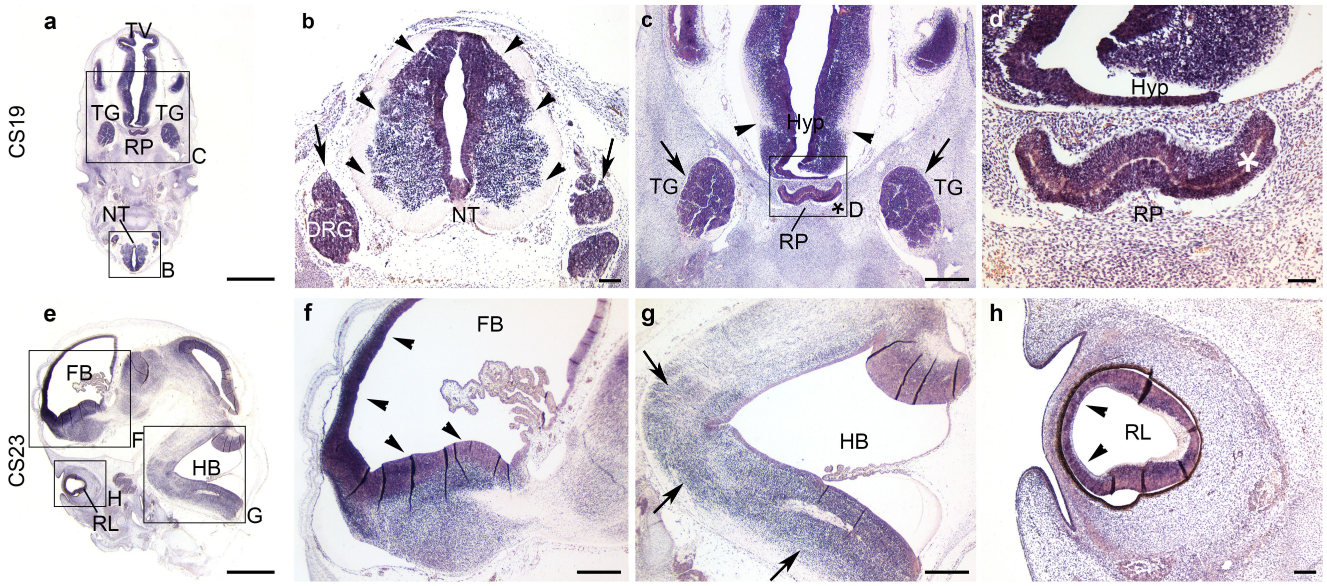

Supplementary Figure 2: *BRAF* is expressed in the hypothalamus and pituitary gland during human embryonic development. *In situ* hybridisation performed on coronal **(a)** or sagittal section **(e)** of human embryos at 6 weeks (Carnegie stage, CS 19) and 8 weeks (CS-23) of human gestation. **(b, c)** and **(f-h)** are higher-magnification views of the boxed areas in **(a)** and **(e)**, respectively. **(d)** Represents higher magnification of boxed area in **(c)**. **(a-d)** At 6 weeks of gestation, *BRAF* is extensively expressed within the neural tube (NT, arrowheads in **b**), the dorsal root ganglia (DRG, arrows in **b**) and the trigeminal ganglia (TG, arrows in **c**). *BRAF* transcripts are localised in the hypothalamus (Hyp, arrowheads in **c)** and in Rathke's pouch, the primordium of the pituitary gland (RP, asterisks in **c** and **d**). **(e-h)** At 8 weeks of gestation, *BRAF* transcripts are localised in the forebrain (FB, arrowheads in **f**) and hindbrain (HB, arrows in **g**). At this stage, the refractive lens has a wide spread expression of *BRAF* (RL, arrowheads in **h**). Images are representative of 3 independent experiments. Abbreviations: NT, neural tube; TG, trigeminal ganglia; Hyp, hypothalamus; RP, Rathke's pouch; FB, forebrain; HB, hindbrain; RL, refractive lens. Scale bars represent: 200 µm **(d)**; 250 µm **(b, c, f, g, h)**; 500 µm **(a, e)**.

Supplementary Figure 3

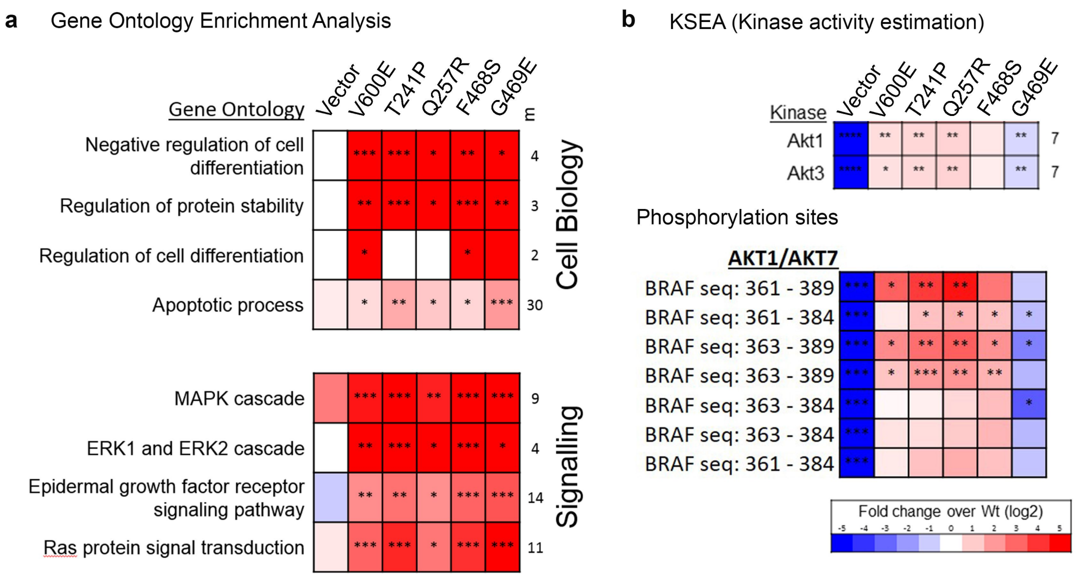

Supplementary Figure 3: Gene ontology enrichment analyses (a) and kinase activity estimation (b) suggest functional pathways downstream of pathogenic BRAF variants, p.T241P, p.Q257R, p.F468S and p.G469E. (a) Gene ontology enrichment identified hyper phosphorylated peptides involved in negative regulation of cell differentiation, apoptotic processes and protein stability by expression of the human BRAF activating mutations (top panel). Pathway analysis revealed that cells transfected with the BRAF variants presented an enrichment in the phosphorylation of proteins linked to the MAPK pathway, ERK/ERK2 pathway, epidermal growth factor receptor pathway and RAS signal transduction components (lower panel). **(b)** Kinase activity identified hyper phosphorylation of Akt1/Akt3 involved in senescence associated with four of the BRAF variants p.V600E, p.T241P, p.Q257R and p.F468S, with weaker hyper-phosphorylation in p.G469E compared to wild type BRAF vector.

Supplementary Figure 4

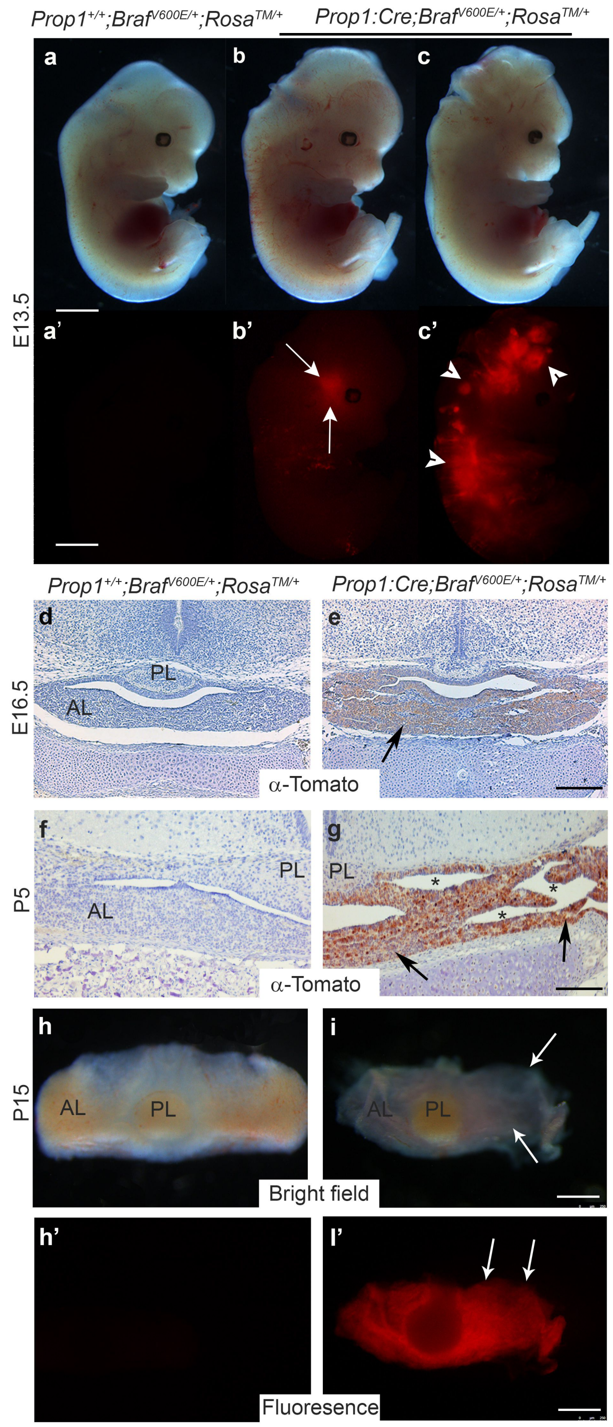

Supplementary Figure 4: **Ectopic expression of Cre recombinase outside of the pituitary gland in the *Prop1:Cre;Braf*$^{V600E/+}$*,Rosa*$^{TM/+}$ (c') is visualised by fluorescent Tomato expression**. 60% of *Prop1:Cre;Braf*$^{V600E/+}$*,Rosa*$^{TM/+}$ embryos exhibit Cre recombinase activity in the pituitary gland as visualised under the fluorescent microscope at E13.5 (white arrows in **b'**) compared to Wt without Tomato expression **(a-a')**. Embryos that exhibited expression of the Tomato in tissues other than the pituitary gland, such as the neural tube or forebrain (white arrowheads embryo in **c'**) were excluded from the study. **(d-g)** IHC for α-Tomato on coronal sections through the pituitary gland of a Wt animal at E16.5 and P5 **(d, f)** compared with *Prop1:Cre;Braf*$^{V600E/+}$*,Rosa*$^{TM/+}$ mutant (**e, g**). Note that Cre activity visualised by expression of Tomato is restricted to the anterior pituitary gland only (**f, g**). **(h, i)** Whole mount pictures of Wt **(h)** and mutant **(i)** pituitaries at P15 reveal severe hypoplasia of the AL (arrows in **i**). **(h', i')** Represent epifluorescent images of the pituitaries from **(h)** and **(i)**, which reveal Tomato expression only in the AL of the pituitary but not in the PL. Images are representative of 4 embryos per genotype. Asterisks in (**g**) denote cavities in the AL. Abbreviations: AL, anterior lobe; E, embryonic day; IHC, immunohistochemistry; PL, posterior lobe. Scale bars in (**a-c**) and (**a'-c'**) represent 0.25 cm, in (**e**) and (**g**) represent 300 μm and in (**i-i'**) 500 μm.

Supplementary Figure 5

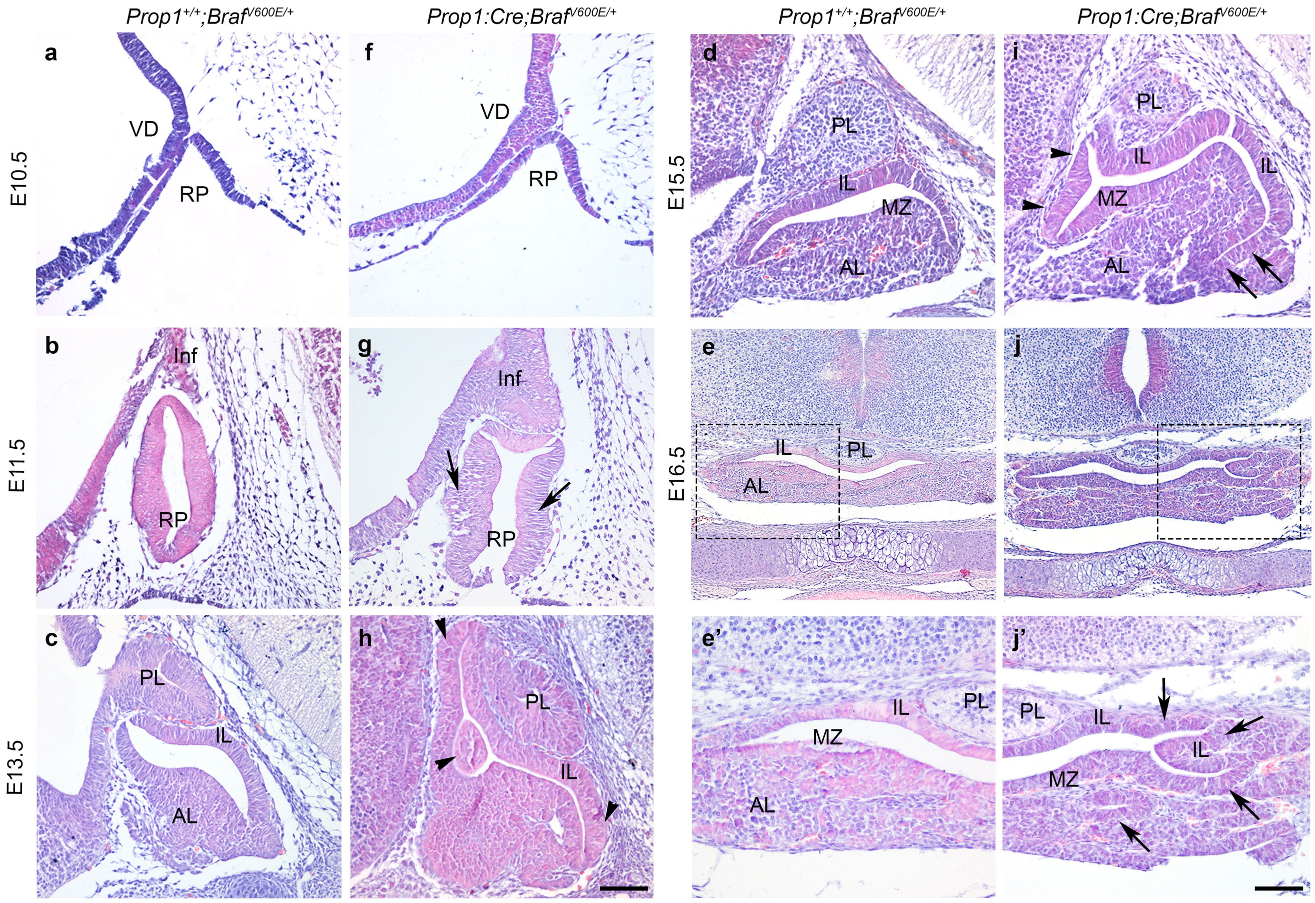

Supplementary Figure 5: *Prop1:Cre;Braf$^{V600E/+}$* embryos exhibit morphological abnormalities in the developing pituitary gland. **(a-j)** Haematoxylin and eosin staining of sagittal (**a-d** and **f-i**) and frontal (**e**, **j**) sections through the developing pituitary gland in Wt (**a-e**) and mutant *Prop1:Cre;Braf$^{V600E/+}$* (**f-j**) embryos. The first apparent phenotypical abnormalities appear at E11.5 with considerable thickening of the lumen of Rathke's Pouch (RP, arrows in **g**) compared to the wild type (**b**). **(c, h)** By E13.5, the *Prop1:Cre;Braf$^{V600E/+}$* mutant pituitaries exhibit gross morphological abnormalities with overgrowth of the dorsal lumen of the RP, the intermediate lobe (IL) in contact with the posterior lobe (PL). This lumen branches and expands dorsally and ventrally (arrowheads in **h**). **(d, i)** At E15.5, three lobes are apparent, posterior lobe (PL), intermediate lobe (IL), anterior lobe (AL) with the epithelial cells lining the cleft or marginal zone (MZ). The *Prop1:Cre;Braf$^{V600E/+}$* mutant pituitaries exhibit an enlargement of the lumen of RP with an increased IL and a large cleft area (MZ) with bifurcations that expand dorsally (**i**, arrowheads) and ventro-caudally (**i**, arrows). **(e, j)** At E16.5, the pituitary gland of the *Prop1:Cre;Braf$^{V600E/+}$* mutant embryos (**j**) exhibit an enlarged MZ and IL which constitute most of the anterior pituitary (arrows in **j'**). The AL parenchyma in the *Prop1:Cre;Braf$^{V600E/+}$* mutant pituitaries is formed of extended folds of IL and MZ (arrows in **j'**) compared to the wild type littermates (**e** and **e'**). **(e' and j')** represent enlarged images of the squared areas in (**e**) and (**j**), respectively. Pictures are representative of 8 embryos per genotype. Abbreviations: E, embryonic day; AL, anterior lobe; IL, intermediate lobe; Inf, Infundibulum; PL, posterior lobe; RP, Rathke's Pouch; VD, ventral diencephalon; MZ marginal zone. Scale bars in (**h**) and (**j'**) represent 200 μm.

Supplementary Figure 6

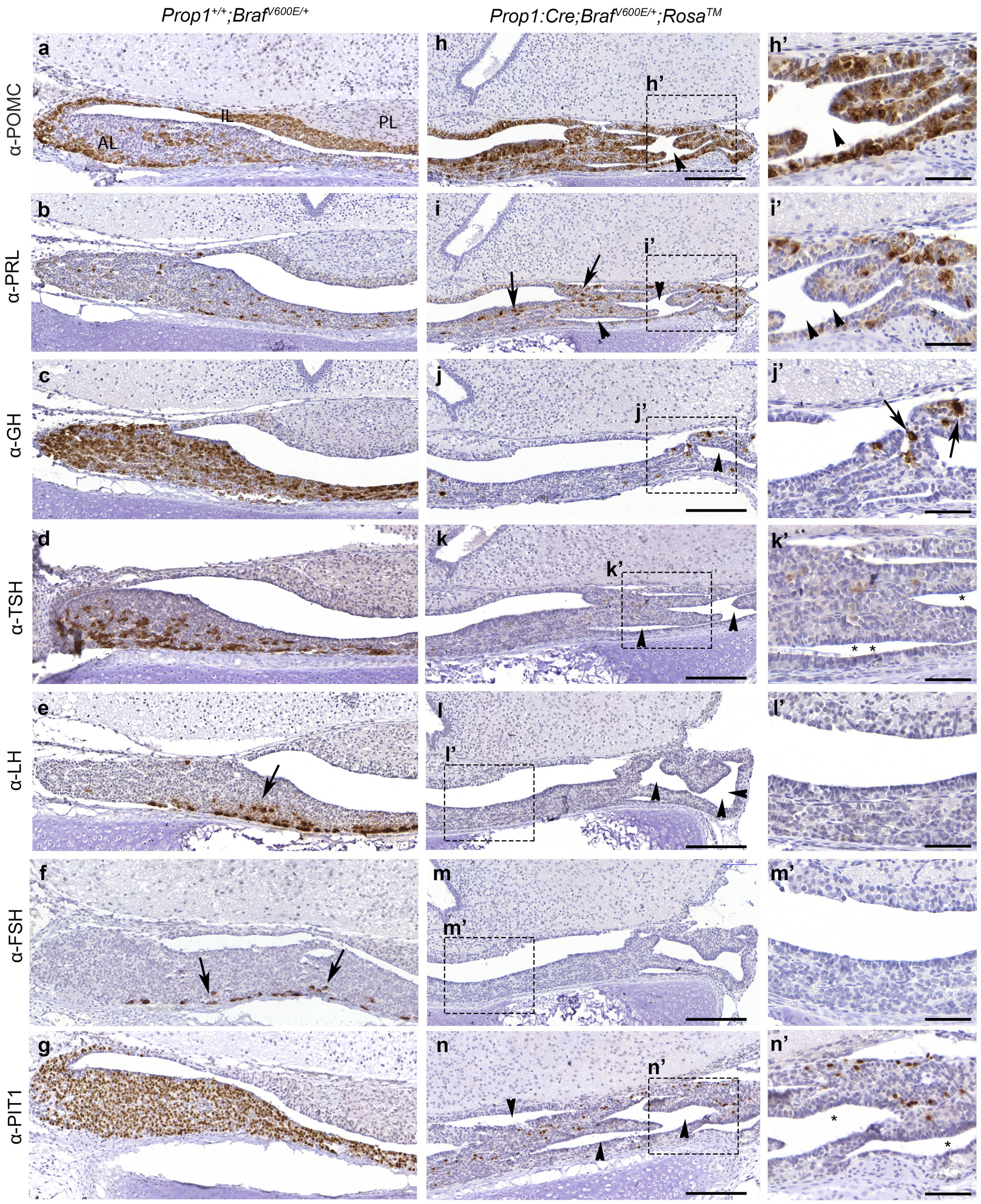

Supplementary Figure 6: Lack of terminal differentiation of TSH-, FSH- and LH+ve and severe reduction of GH+ve cells in the *Prop1:Cre;Braf$^{V600E/+}$;Rosa$^{TM/+}$* mutant pituitaries at P5. Immunohistochemistry against hormone-producing cells in Wt (**a-f**) and *Prop1:Cre;Braf$^{V600E/+}$;Rosa$^{TM/+}$* (**h-n**) reveals increased expression of POMC (**a, h**) and PRL (**b, i**) in mutant pituitaries at P5 compared to Wt littermates. **(j)** GH+ve cells were severely reduced in *Prop1:Cre;Braf$^{V600E/+}$;Rosa$^{TM/+}$* mutants compared to their Wt littermates (**c**) with only a few positive scattered cells (arrows in **j'**). TSH (**d**, **k**), LH (**e**, **l**) and FSH (**f**, **m**) were completely absent in the *Prop1:Cre;Braf$^{V600E/+}$;Rosa$^{TM/+}$* pituitaries compared to Wt littermates. **(g, n)** Expression of the cell lineage transcription factor Pit1 was severely reduced in mutant pituitaries (**n**, **n'**) compared to Wt (**g**). Note that mutant pituitaries *Prop1:Cre;Braf$^{V600E/+}$;Rosa$^{TM/+}$* (**h-n**) exhibit severe hypoplasia compared to Wt (**a-g**), and are formed of a thin layer of cells with a multitude of cavities within the parenchyma arrowheads and asterisks in (**h, h', i, i', j, k, k', l, n, n'**) . **(h'-n')** represent magnified images from the squared areas in (**h-n**). Asterisks in **k'** and **n'** represent tissue cavities in the AL parenchyma. Images are representative of n=5 embryos per genotype. Abbreviations: AL, anterior lobe; IL, intermediate lobe; PL, posterior lobe. Scale bars in (**h-n**) represent 200 µm and in (**h'-n'**) represent 50 µm.

Supplementary Figure 7

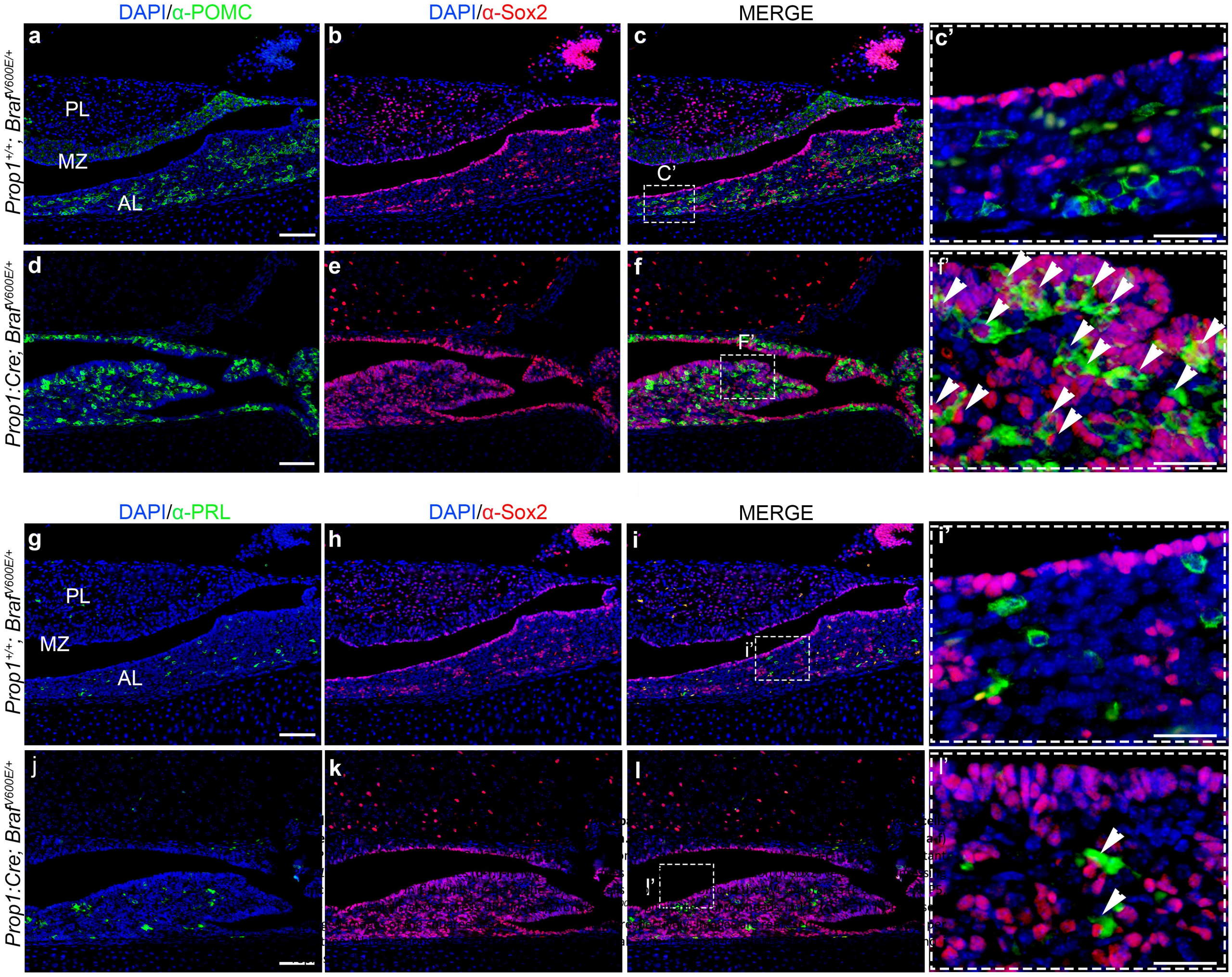

Supplementary Figure 7: Activation of the ERK/MAPK pathway results in a proportion of Sox2+ve stem cells expressing Pomc and PRL that are maintained after birth. Double immunofluorescence against Pomc (green, **a-f**) or PRL (green, **g-l**), and Sox2 (red, **a-l**) in coronal sections at P5 of Wt (**a-c**; **g-i**) and mutant *Prop1:Cre;Braf*$^{V600E/+}$ pituitaries (**d-f**; **j-l**). Mutant pituitaries exhibit a large proportion of Sox2+ve cells co-expressing Pomc (arrowheads in **f'**), whilst none of the Sox2+ve cells expressed Pomc in the Wt pituitaries (**c** and **c'**). At P5, some Sox2+ve cells also express PRL in *Prop1:Cre;Braf*$^{V600E/+}$ pituitaries (arrowheads in **l'**). **(c', f', i', l')** represent enlarged images of the squared areas in (**c**), (**f**), (**i**) and (**l**) respectively. Images are representative of 3 embryos per genotype. Abbreviations: AL, anterior lobe; MZ, marginal zone; PL, posterior lobe. Scale bars in **a**, **d**, **g** and **j** represent 150 µm and in **c'**, **f'**, **i'** and **l'** represent 40 µm.

Supplementary Figure 8

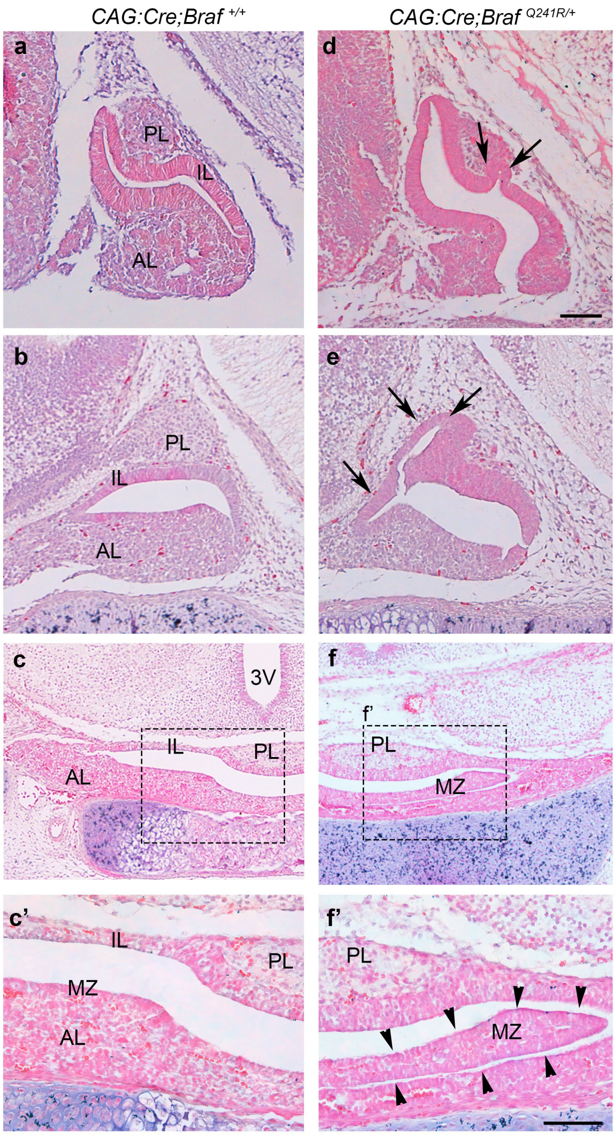

Supplementary Figure 8: Expression of the *Braf*$^{Q241R/+}$ allele results in morphological abnormalities of the pituitary gland. **(a-f)** Haematoxylin and eosin staining of sections through the pituitary gland of Wt (**a-c**) and mutant *CAG:Cre;Braf*$^{Q241R/+}$ (**d-f**) at E13.5 (**a**, **d**); E15.5 (**b**, **e**) and E18.5 (**c**, **f**) revealed morphological abnormalities. At E13.5 the pituitary cleft and intermediate lobe exhibit overgrowths (arrows in **d**) compared to Wt (**a**). By E15.5 *CAG:Cre;Braf*$^{Q241R/+}$ mutant pituitary glands exhibit abnormal MZ with apparent overgrowth and bifurcations (arrows in **e**). At E18.5 overgrowths result in extended MZ within the pituitary lumen (arrowheads in **f'**) and a hypoplastic AL. **(c'**, **f')** represent higher magnification of the boxed areas in (**c, f**) respectively. Images are representative of 5 embryos per genotype. Abbreviations: AL, anterior lobe; IL, intermediate lobe; MZ, marginal zone; PL, posterior lobe; 3V third ventricle. Scale bar in (**d**) represents 50 µm and in (**f'**) represents 200 µm.

Supplementary Figure 9

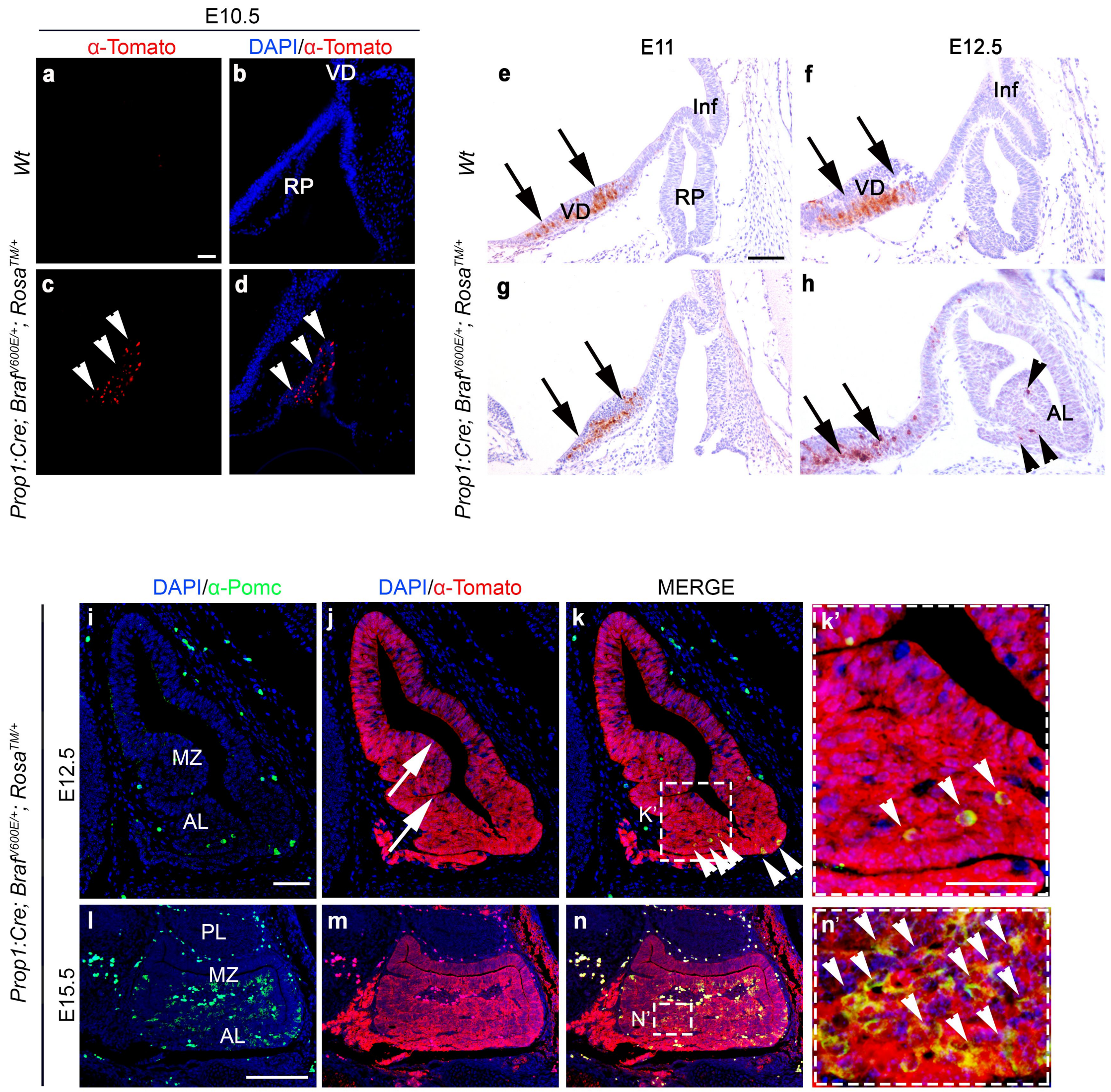

Supplementary Figure 9: The Cre recombinase activity of the *Prop1:Cre* transgenic line starts early during RP development and marks Pomc cells. At E10.5, immunostaining with α-Tomato (red, **a-d**) reveals expression of Tomato in scattered cells along the developing RP of *Prop1:Cre;Braf$^{V600E/+}$;Rosa$^{TM/+}$* embryos (white arrowheads in **c** & **d**). Immunostaining on sagittal sections of E11 (**e**, **g**) and E12.5 (**f**, **h**) using α-Pomc antibody reveals appearance of Pomc+ve cells in the ventral diencephalon at E11 (VD, arrows in **e** & **g**) but not in the RP (**e** & **g**). At E12.5, a few Pomc positive cells appear in the ventral part of the anterior lobe (AL) of *Prop1:Cre;Braf$^{V600E/+}$;Rosa$^{TM/+}$* mutant pituitaries (arrowheads in **h**). Double immunofluorescence on sagittal sections of *Prop1:Cre;Braf$^{V600E/+}$;Rosa$^{TM/+}$* mutant pituitaries with α-Tomato and α-Pomc antibodies at E12.5 (**i-k**) and E15.5 (**l-n**) reveal co-expression between Tomato+ve cells and Pomc -expressing cells, indicating that Cre activity also occurs in the emerging Pomc cells of mutant pituitaries (arrowheads in **k**, **k'** and **n'**). **(k' & n')** represent enlarged images of squared areas in (**k**) and (**n**), respectively. Images are representative of 3 embryos per genotype. Note, abnormal overgrowth of the cleft in mutant pituitaries (arrows in **j**). Abbreviations: AL, anterior lobe; Inf, infundibulum; MZ, marginal zone; PL, posterior lobe; RP, Rathke's Pouch; VD; ventral diencephalon. Scale bars in (**a**) and (**l**) represent 200 µm, in (**e**) and (**i**) represent 100 µm. Scale bar in (**k'**) represents 50µm.

Supplementary Figure 10

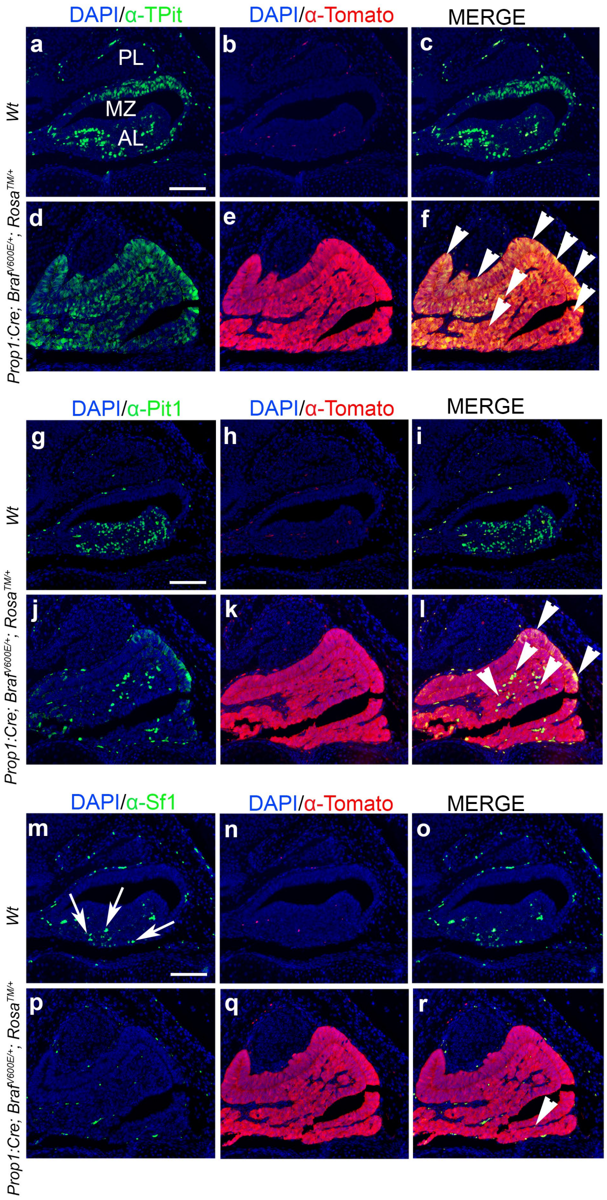

Supplementary Figure 10: Genetic lineage tracing of the *Prop1:Cre;Braf$^{V600E/+}$;Rosa$^{TM/+}$* line reveals Tomato labelling of all the emerging pituitary committed lineages : TPit, Pit1 and Sf1. Double immunofluorescence against α-Tomato (red, **a**-**r**) and α-TPit (green, **a**-**f**), α-Pit1 (green, **g**-**l**) or α-Sf1 (green, **m**-**r**) on sagittal sections at E15.5 of Wt (**a**-**c**; **g**-**i**; **m**-**o**) and *Prop1:Cre;Braf$^{V600E/+}$;Rosa$^{TM/+}$* mutant pituitaries (**d**-**f**; **j**-**l**; **p**-**r**). At E15.5 all *Prop1:Cre;Braf$^{V600E/+}$;Rosa$^{TM/+}$* cells in the anterior pituitary gland express Tomato (**e**, **k**, **q**) from the *Rosa^{CAGLxpSTOPLxpTomato}* locus, indicating that the Cre recombinase marks all the descendants of the anterior pituitary gland. Tomato positive cells co-express the pituitary commitment markers TPit (arrowheads in **f**), Pit1 (arrowheads in **l**) and Sf1 positive cells (arrowhead in **r**). Note a significant increase in TPit cells (**d**) in *Prop1:Cre;Braf$^{V600E/+}$;Rosa$^{TM/+}$* and a decrease in Pit1 (**j**) and Sf1 (**p**), compared to Wt (**a**, **g**, arrows in **m**, respectively). Images are representative of 3 embryos per genotype. Abbreviations: AL, anterior lobe; MZ, marginal zone; PL, posterior lobe. Scale bars in (**a**, **g** & **m**) represent 200 µm.

Supplementary Figure 11

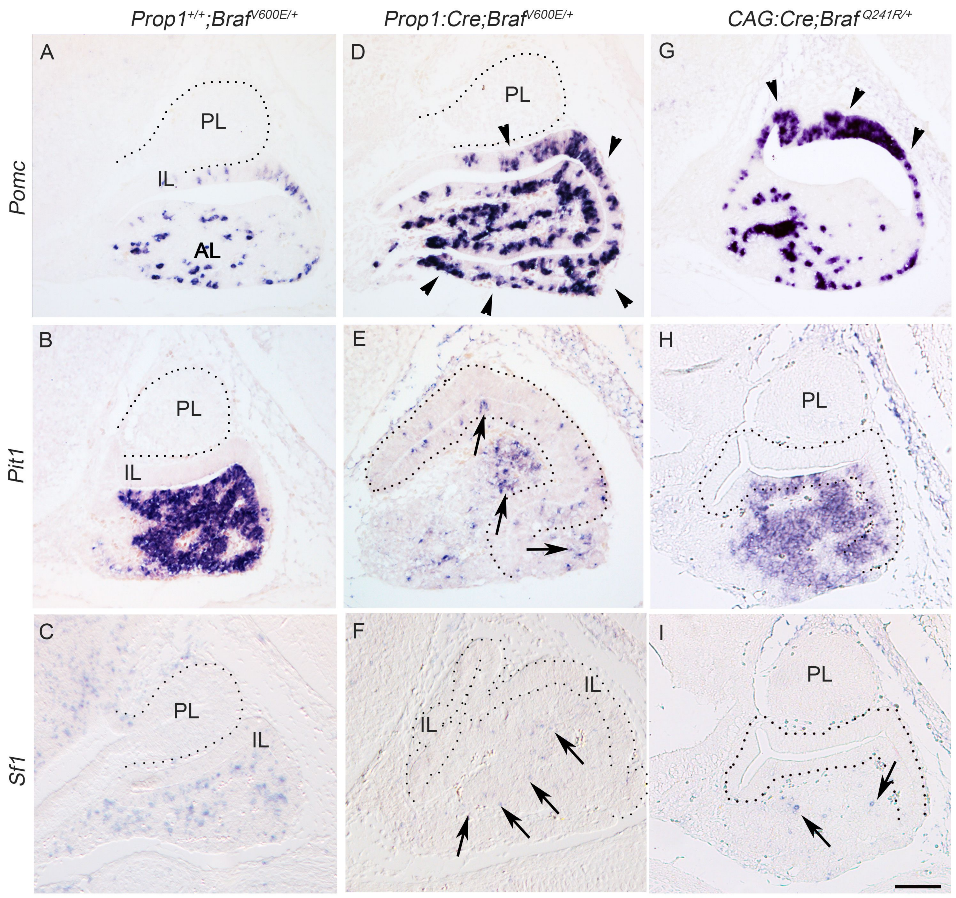

Supplementary Figure 11: Expression of *Braf*^{V600E} and *Braf*^{Q241R} lead to the abnormal determination of the cell lineage factors *Pomc, Pit1* and *Sf1*. **(a-i)** *In situ* hybridisation for *Pomc* (**a**, **d**, **g**), *Pit1* (**b**, **e**, **h**) and *Sf1* (**c**, **f**, **i**) on sagittal sections of E15.5 Wt (**a-c**), *Prop1:Cre;Braf*$^{V600E/+}$ (**d-f**) and *CAG:Cre;Braf*$^{Q241R/+}$ (**g-i**) embryos. *Prop1:Cre;Braf*$^{V600E/+}$ pituitaries exhibit an upregulation in *Pomc* mRNA transcripts (**d**) compared to the Wt littermates (**a**). A milder increase in *Pomc* transcripts was also observed in *CAG:Cre;Braf*$^{Q241R/+}$ pituitaries (**g**) compared to Wt (**a**). The expression of Pit1 in *Prop1:Cre;Braf*$^{V600E/+}$ pituitaries was found to be severely reduced by *in situ* hybridisation (arrows in **e**) and lower levels of Pit1 expression were also observed in the *CAG:Cre;Braf*$^{Q241R/+}$ pituitaries (**h**). *Sf1* mRNA transcripts were found to be downregulated in both *Prop1:Cre;Braf*$^{V600E/+}$ and *CAG:Cre;Braf*$^{Q241R/+}$ pituitaries (arrows in **f** and **i**, respectively) compared to Wt (**c**). Note that the mutant pituitary glands are composed of an expanded and bifurcated IL (arrowheads in **d-f** and **g-h**) although morphological abnormalities were more severe in *Prop1:Cre;Braf*$^{V600E/+}$ mutant pituitaries compared to *CAG:Cre;Braf*$^{Q241R/+}$. Abbreviations: AL, anterior lobe; IL, intermediate lobe; PL, posterior lobe. Images are representative of n=5 embryos per genotype. Scale bar in (**i**) represents 200 µm.

Supplementary Figure 12

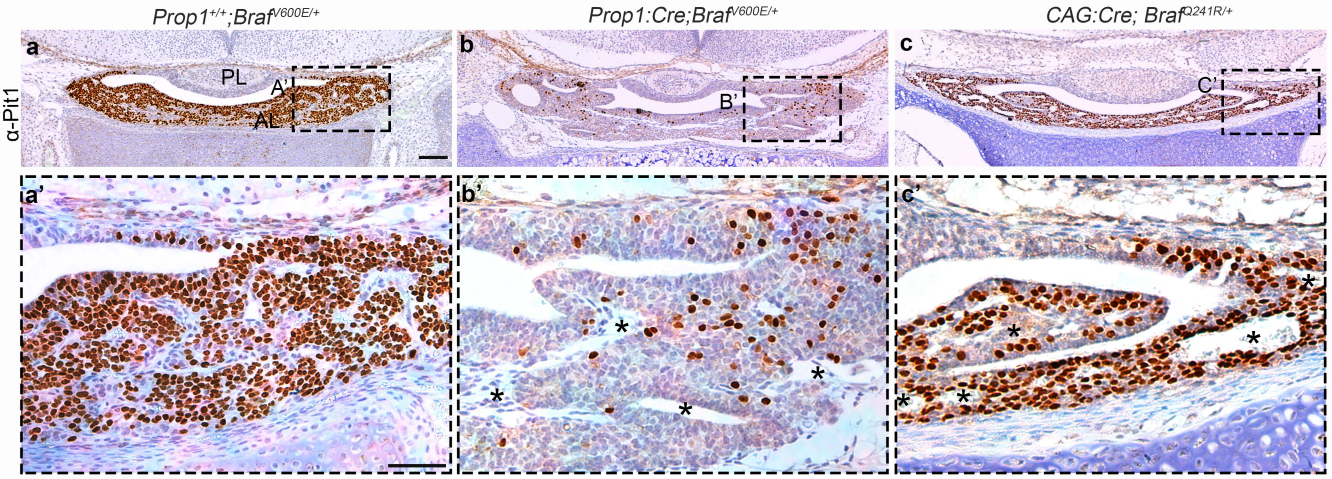

Supplementary Figure 12: Expression of *Braf*^{V600E} leads to severe decrease in Pit1 expression in *Prop1:Cre;Braf*$^{V600E/+}$ pituitaries and small reduction of Pit1 in *CAG:Cre;Braf*$^{Q241R/+}$ pituitaries compared to Wt. **(a-c)** Coronal sections through the anterior pituitary gland at E18.5 of Wt (**a**), *Prop1:Cre;Braf*$^{V600E/+}$ (**b**) and *CAG:Cre;Braf*$^{Q241R/+}$ (**c**) immunostained against α-Pit1. The number of Pit1+ve cells was severely reduced in the *Prop1:Cre;Braf*$^{V600E/+}$ (**b**, **b'**) compared to Wt (**a**). A milder reduction of Pit1+ve cells was observed in *CAG:Cre;Braf*$^{Q241R/+}$ (**c**) compared to Wt. Tissue cavities within the parenchyma of the AL were visible in both Prop1:Cre;BrafV$^{600E/+}$ and *CAG:Cre;Braf*$^{Q241R/+}$ compared to Wt (asterisks in **b'** and **c'**). Abbreviations: AL, anterior lobe; PL, posterior lobe. Scale bar in (**a**) represents 250 µm. Scale bar in (**a'**) represents 100 µm.

Supplementary Figure 13

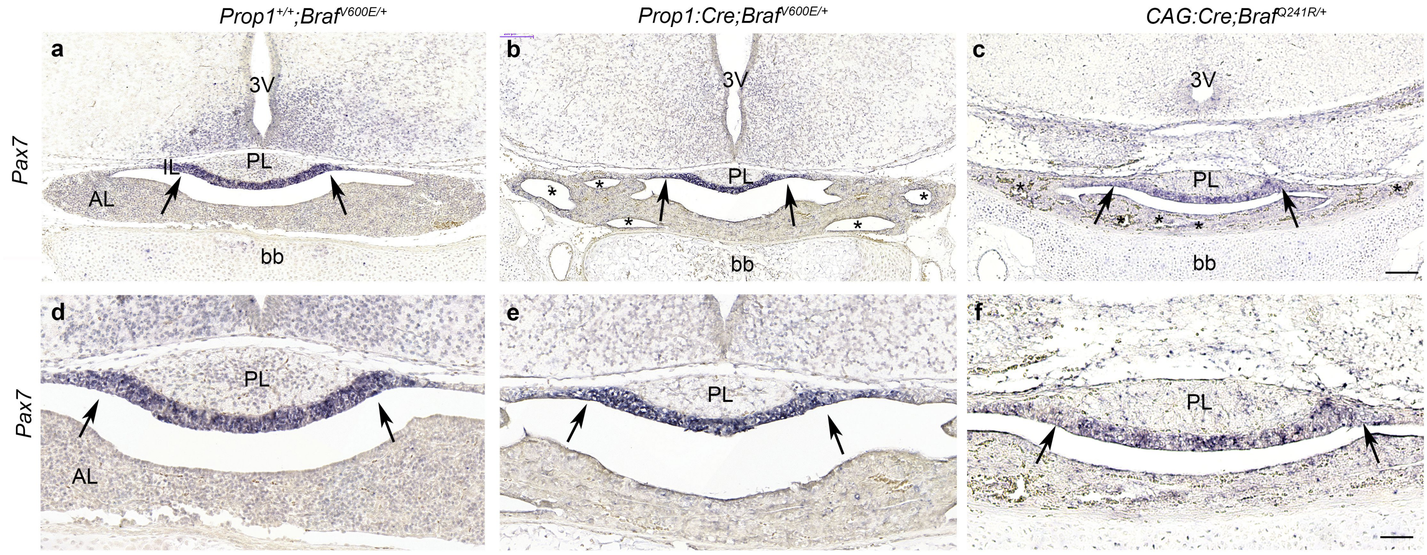

Supplementary Figure 13: The intermediate lobe marker *Pax7* that specifies melanotrophs is normally expressed in both *Prop1:Cre;Braf$^{V600E/+}$* and the *CAG:Cre;Braf$^{Q241R/+}$* mutant pituitaries. **(a-f)** Coronal sections through the pituitary gland of P1 Wt (**a**, **d**), *Prop1:Cre;Braf$^{V600E/+}$* (**b**, **e**) and *CAG:Cre;Braf$^{Q241R/+}$* (**c**, **f**) pituitary glands hybridised with *Pax7* mRNA antisense probe. *Pax7* mRNA transcripts were found to be expressed in the intermediate lobe (IL) in both Wt and mutant pituitaries (arrows in **a**-**f**), indicating that the activation of MAPK pathway by the expression of *Braf^{V600E}* or *Braf^{Q241R}* does not alter the domain of expression of the melanotroph lineage restriction marker *Pax7*. Note that the mutant pituitaries *Prop1:Cre;Braf$^{V600E/+}$* and *CAG:Cre;Braf$^{Q214R/+}$* exhibited multiple cavities indicative of tissue degeneration (asterisks in **b** and **c**). Images are representative of 4 embryos per genotype. Abbreviations: AL, anterior lobe; bb, basisphenoid bone; IL, intermediate lobe; PL, posterior lobe; 3V third ventricle. Scale bar in (**c**) represents 150 µm and in (**f**) represents 75 µm.

Supplementary Figure 14

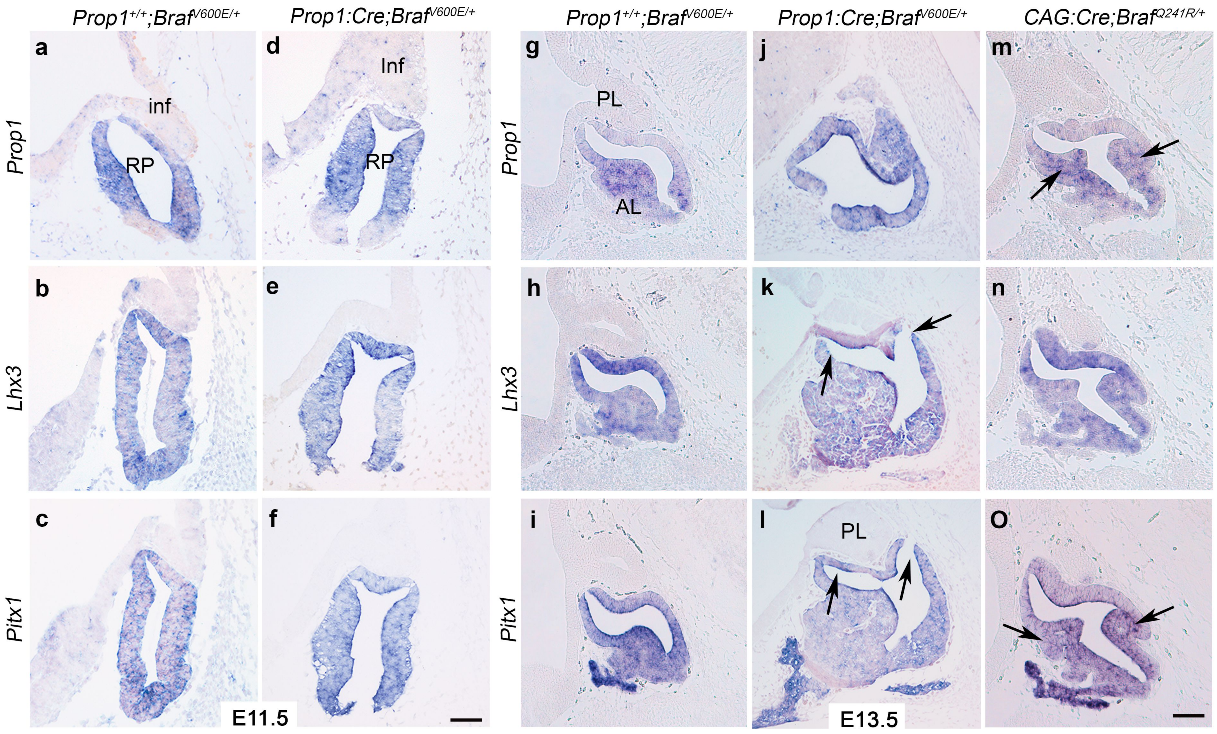

Supplementary Figure 14: Activation of the ERK/MAPK pathway does not affect early pituitary specification. **(a-o)** *In situ* hybridisation for the early pituitary transcription factors *Prop1*, *Lhx3* and *Pitx1* on sagittal sections of E11.5 (**a-f**) and E13.5 (**g-o**) embryos. At E11.5, *Prop1*, *Lhx3* and *Pitx1* mRNA transcripts are normally expressed in the developing RP in both Wt (**a-c**) and *Prop1:Cre;Braf$^{V600E/+}$* (**d-f**). The expression pattern of these transcription factors is maintained at E13.5 with no apparent differences between Wt (**g-i**), *Prop1:Cre;Braf$^{V600E/+}$* (**j-l**) and *CAG:Cre;Braf$^{Q241R/+}$* (**m-o**) mutant pituitaries. Note that the only difference, at these stages, was morphological with apparent bifurcations and increased cleft area of RP in *Prop1:Cre;Braf$^{V600E/+}$* mutant pituitaries compared to Wt (arrows in **k-l**). *CAG:Cre;Braf$^{Q241R/+}$* mutant pituitaries exhibited a milder phenotype (arrows in **m-o**). Images are representative of 4 embryos per genotype. Abbreviations: Inf, infundibulum; RP, Rathke's Pouch; AL, anterior lobe; PL, posterior lobe. Scale bar in (**f**) represents 50 µm and in (**o**) represents 100 µm.

Supplementary Figure 15

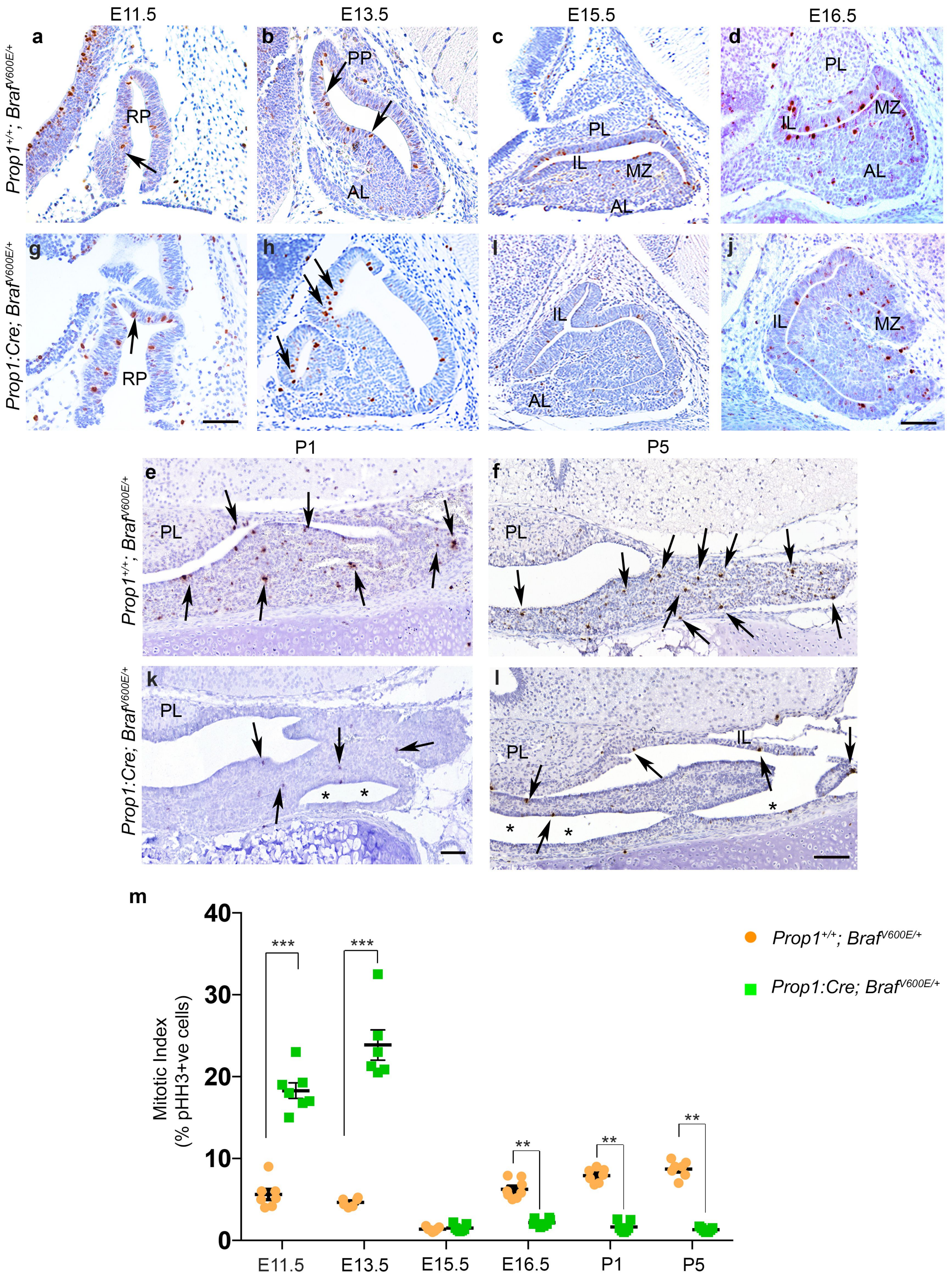

Supplementary Figure 15: Expression of *Braf*$^{V600E/+}$ in the pituitary causes a transient increase in proliferation of the pituitary progenitor cells with a subsequent decline of proliferation and pituitary growth arrest over time. **(a-l)** Immunohistochemistry against phospho-histone H3 (α-pHH3) in wild type (**a-f**) and *Prop1:Cre;Braf*$^{V600E/+}$ pituitaries (**g-l**). Note that most of the α-pHH3 positive cells are located in the lumen of the RP (arrows in **a**, **b**, **g** and **h**). **(m)** Quantitative analysis of the mitotic index (MI) shows that *Prop1:Cre;Braf*$^{V600E/+}$ pituitaries have a significantly higher MI at E11.5 and E13.5 compared to the Wt littermates, but this is transient and the proliferation rate is comparable between genotypes at E15.5. The proliferation capacity of the mutant *Prop1:Cre;Braf*$^{V600E/+}$ pituitaries significantly declines from E16.5 and at postnatal days P1 and P5 compared to Wt (** $p<0.05$; ***$p<0.001$ unpaired two-tailed Student's T-test. Data represented as mean ± SEM from n= 6 or 8 embryos per genotype of each developmental stage). Abbreviations: E, embryonic day; AL, anterior lobe; IL, intermediate lobe; MZ, marginal zone; PL, posterior lobe; RP, Rathke's Pouch. Scale bars in (**g**) represents 50 µm, and in (**j, k & l**) 100 µm.

Supplementary Figure 16

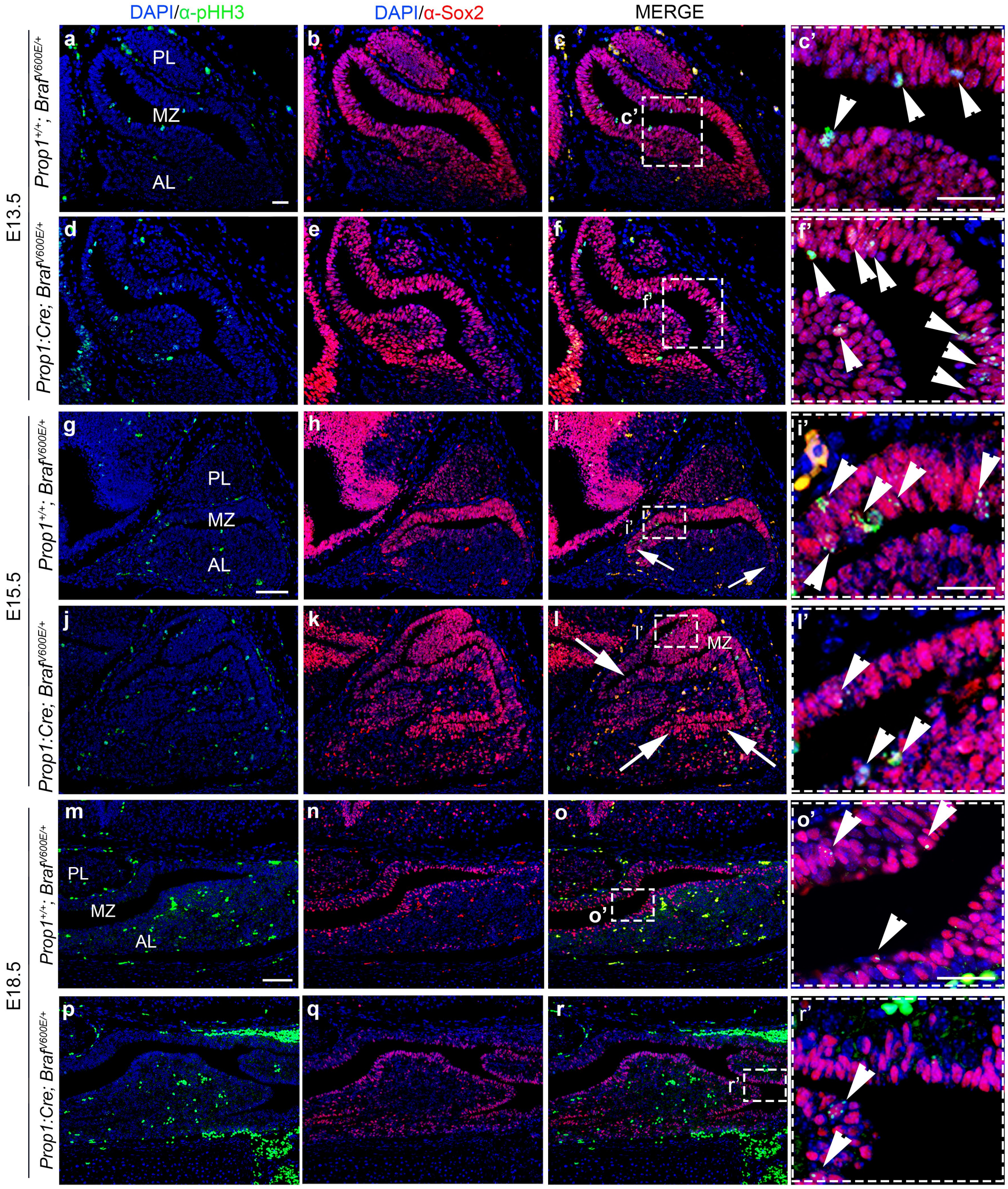

Supplementary Figure 16: The proliferating cells in the *Prop1:Cre;Braf*$^{V600E/+}$ mutant pituitaries are Sox2 positive. Double immunofluorescence against pHH3 (green) and Sox2 (red) at E13.5 (**a-f**); E15.5 (**g-l**) and E18.5 (**m-r**) reveals co-localisation of pHH3 positive cells with the pituitary stem cell marker Sox2 (white arrowheads in **c'** & **f'**; **i'**& **l'**; **o'**& **r'**). A big increase in the number of double pHH3+ve and Sox2+ve cells in the marginal zone (MZ) was observed at E13.5 in mutant pituitaries (white arrowheads in **f'**) compared to Wt (**c'**). Increased proliferation of the Sox2+ve cells leads to an enlargement of the stem cell compartment by E15.5 in the mutant (white arrows in **l**) compared to Wt pituitaries (white arrows in **i**). A similar number of double pHH3-Sox2+ve cells were seen in E15.5 mutant pituitaries compared to WT (arrowheads in **l'** compared to **i'**) indicating a similar mitotic index at this stage. By E18.5, double pHH3-Sox2+ve cells were mainly found within the MZ, but at this specific gestational stage, less pHH3+ve cells were observed in mutant pituitaries compared to Wt (arrows in **r'** compared to **o'**). Images are representative of 3 embryos per genotype. (**c'**, **f'**, **i'**, **l'**, **o'** and **r'**) are higher magnification images of the squared areas in (**c**, **f**, **i**, **l**, **o** & **r**) respectively. Abbreviations: AL, anterior lobe; MZ, marginal zone; PL, posterior lobe. Scale bar in (**a**) represents 100 µm. Scale bars in (**g**) and (**m**) represent 200 µm and in (**c'**), (**i'**) and (**o'**) represent 50 µm.

Supplementary Figure 17

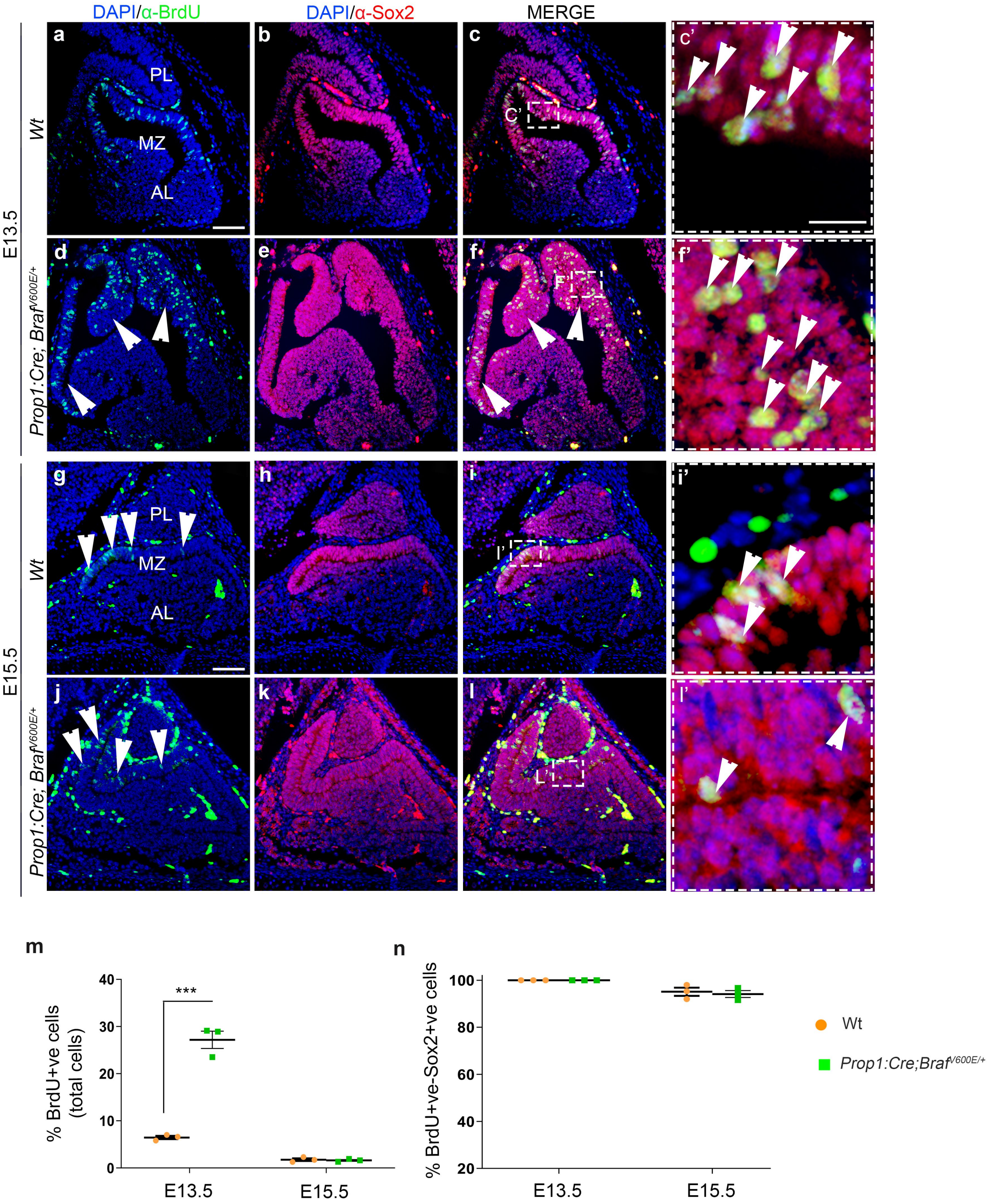

Supplementary Figure 17: BrdU incorporation shows that the proliferating cells in the *Prop1:Cre;Braf*$^{V600E/+}$ mutant pituitaries are Sox2+ve. Double immunofluorescence against BrdU (green) and Sox2 (red) at E13.5 (**a**-**f**) and E15.5 (**g**-**l**) of Wt (**a**-**c** & **g**-**i**) and mutant pituitaries (**d**-**f** & **j**-**l**). More BrdU incorporation along the MZ was detected at E13.5 in mutant pituitaries compared to Wt at E13.5 (arrowheads in **d** and **f**). Quantification of the percentage of BrdU positive cells (**m**) indicates significant increase in BrdU incorporation at E13.5 (***p=0.0004). At E15.5, no difference in % of BrdU positive cells was observed in the mutant compared to Wt pituitaries (arrowheads in **j** and **g**) and quantification (**m**) (p=0.65). At E13.5, 100% of the BrdU+ve cells co-localises with Sox2 (arrowheads in **c'**, **f'**, **i'** and **l'**) in both mutant and Wt pituitaries (**n**). At E15.5 no difference in percentage of BrdU+ve and Sox2+ve cells was observed between genotypes (p=0.688). Images are representative of 3 embryos per genotype and 3 independent experiments. P values calculated using an unpaired two-tailed Student's T-test and data represented as mean ± SEM from n=3 pituitaries per genotype. Abbreviations: AL, anterior lobe; MZ, marginal zone; PL, posterior lobe. Scale bar in (**a**) represents 100 μm, in (**g**) represents 200 μm and in (**c'**) represents 25 μm.

Supplementary Figure 18

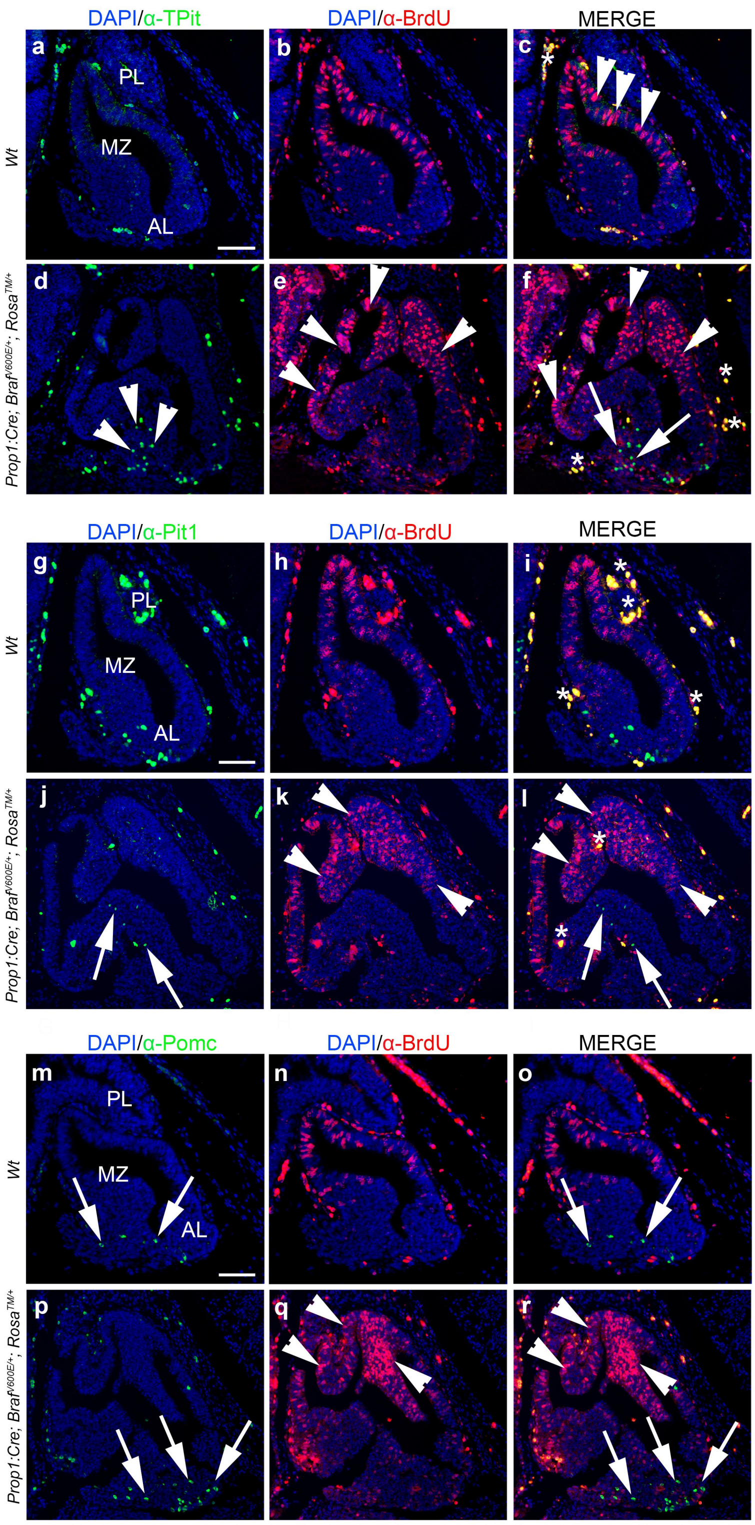

Supplementary Figure 18: The emerging pituitary lineage committed cells TPit, Pit1 and Pomc do not over-proliferate. Double immunofluorescence against BrdU (red, **a**-**r**) and TPit (green, **a**-**f**), Pit1 (green, **g**-**l**) and Pomc (green, **m**-**r**) in E13.5 Wt (**a**-**c**; **g**-**i**, **m**-**o**) and *Prop1:Cre;Braf*$^{V600E/+}$ pituitaries (**d**-**f**, **j**-**l**, **p**-**r**). BrdU staining was detected in the area where the progenitors/PSC reside (arrowheads in **c** and **f**). TPit-, Pit1- and Pomc-expressing cells did not show co-localisation with BrdU, indicating that at this stage these cell types are not proliferative. Note that a big increase in BrdU incorporation in E13.5 mutant pituitary (arrowheads in **e**, **f**, **k**, **l**, **q**, **r**). An increased number of Pomc positive cells was observed in the ventral side of mutant pituitaries compared to Wt (arrows in **p** & **r** compared to arrows in **m** and **o**) indicating that Pomc differentiation is favoured. Note that the red blood cells exhibit auto fluorescence (asterisks). Images are representative of 3 embryos per genotype. Abbreviations: AL, anterior lobe; MZ, marginal zone; PL, posterior lobe. Scale bars in (**a**, **g** & **m**) represent 100 µm.

Supplementary Figure 19

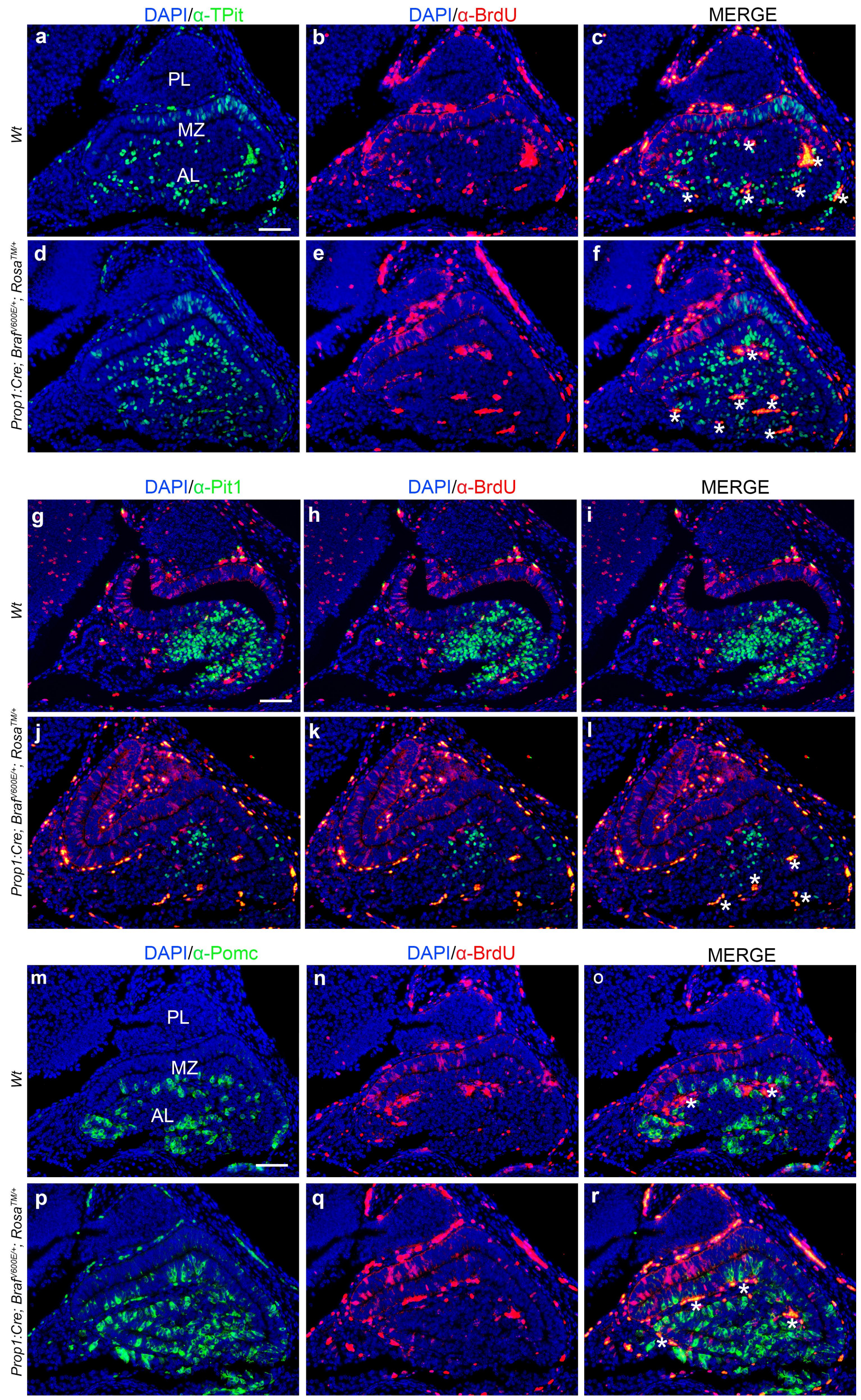

Supplementary Figure 19: **TPit+ve and Pomc+ve cells in *Prop1:Cre;Braf$^{V600E/+}$* mutant pituitaries do not have more proliferative capacity**. Double immunofluorescence against BrdU (red, **a**-**r**) and TPit (green, **a**-**f**), Pit1 (green, **g**-**l**) and Pomc (green **m**-**r**) in Wt (**a**-**c**; **g**-**i**, **m**-**o**) and *Prop1:Cre;Braf$^{V600E/+}$* pituitaries (**d**-**f**, **j**-**l**, **p**-**r**) at E15.5. Most of the BrdU positive cells do not co-express the cell lineage commitment marker TPit, Pit1 and Pomc, indicating that these cell lineages are not over-proliferating in the mutant pituitaries. Note that the red blood cells exhibit autofluorescence (asterisks). Images are representative of 3 embryos per genotype. Abbreviations: AL, anterior lobe; MZ, marginal zone; PL, posterior lobe. Scale bars in (**a**, **g** & **m**) represent 200 µm.

Supplementary Figure 20

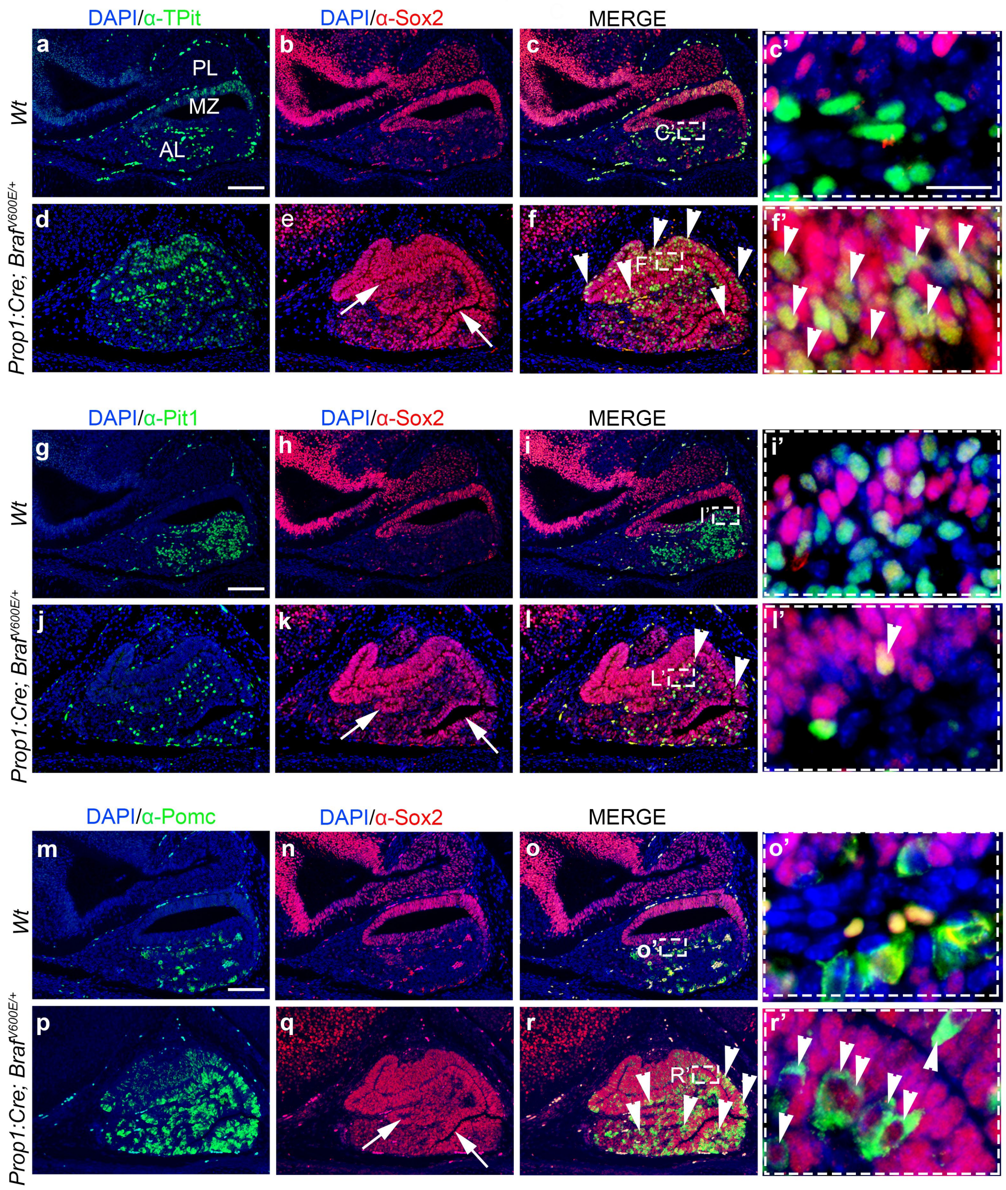

Supplementary Figure 20: Double immunofluorescence of Sox2 with TPit, Pit1 and Pomc in *Prop1:Cre;Braf*$^{V600E/+}$ pituitaries reveals a significant increase in double Sox2-TPit+ve and Sox2-Pomc+ve cells compared to Wt. Sagittal sections through the pituitary gland at E15.5 of Wt (**a**-**c**; **g**-**i**; **m**-**o**) and mutant embryos (**d**-**f**; **j**-**l**; **p**-**r**) stained with α-Sox2 (red, **a**-**r**), α-TPit (green, **a**-**f**), α-Pit1 (green, **g**-**l**) and α-Pomc (green, **m**-**r**). An increase in TPit+ve cells was observed in the *Prop1:Cre;Braf*$^{V600E/+}$ mutant pituitaries (**d**) compared to Wt (**a**). Merged images revealed a high numbers of double TPit-Sox2 positive cells (arrowheads in **f**-**f'**) compared to Wt (**c**-**c'**). Note that the expanded marginal zone with an increase in Sox2+ve cells was observed in *Prop1:Cre;Braf*$^{V600E/+}$ mutant pituitaries (arrows in **e**, **k**, **q**). Pit1 was considerably reduced in mutant pituitaries (**j**) compared to Wt (**g**), with very few double Pit1-Sox2+ve cells (arrows in **l**-**l'**). Pomc cells appeared to be significantly increased in *Prop1:Cre;Braf*$^{V600E/+}$ pituitaries (**p**) compared to Wt (**m**). Merged images revealed co-expression of Sox2+ve cells with Pomc (arrowheads in **r** and **r'**), whilst no co-localisation was observed in Wt (**o**). (**c', f', i', l', o', r'**) represent enlarged images of the square areas in (**c**, **f**, **i**, **l**, **o**, **r**) respectively. Images are representative of 3 embryos per genotype. Abbreviations: AL, anterior lobe; MZ, marginal zone; PL, posterior lobe. Scale bars in (**a**, **g** & **m**) represent 200 μm.

Supplementary Figure 21

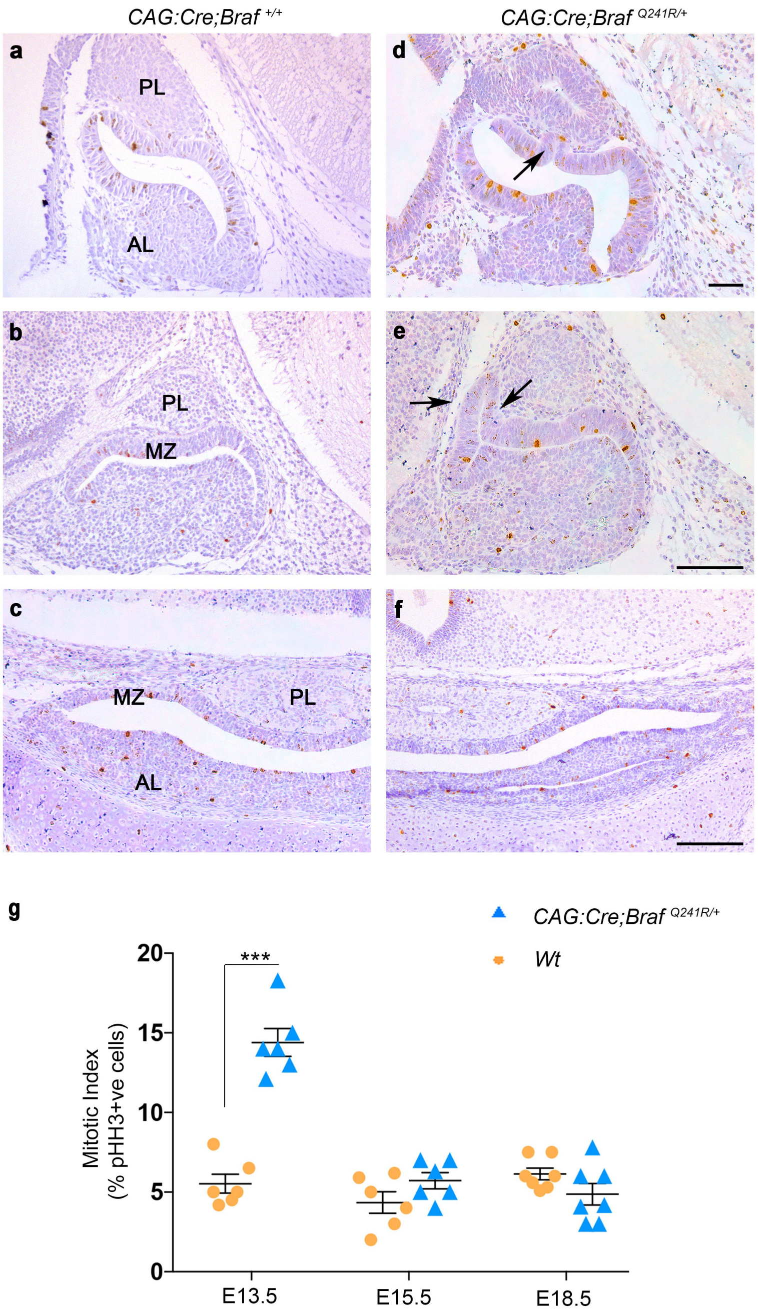

Supplementary Figure 21: Expression of *Braf*^{Q241R} leads to transient increase in mitotic index. (a-f) Immunohistochemistry against phospho-histone H3 (α-pHH3) in Wt (**a**-**c**) and *CAG:Cre;Braf*$^{Q241R/+}$ pituitaries (**d**-**f**). **(g)** Quantitative analysis of the mitotic index (MI) shows that *CAG:Cre;Braf*$^{Q241R/+}$ pituitaries have a significantly higher MI at E13.5 compared to the Wt littermates, but this is a transient effect and the proliferation rate is comparable between genotypes at E15.5 and E18.5 with a trend to less proliferation at E18.5 although not statistically significant (p=0.12). (*** p<0.001; unpaired two-tailed Student's T-test. Data represented as mean ± SEM from n=6/7 embryos per genotype per developmental stage). The MI represents the percentage of pHH3+ve cells over total amount of cells. Abbreviations: E, embryonic day; AL, anterior lobe; IL, intermediate lobe; MZ, marginal zone; PL, posterior lobe. Scale bars in (**d**) represents 50 µm, in (**e**) and (**f**) represent 200 µm.

Supplementary Figure 22

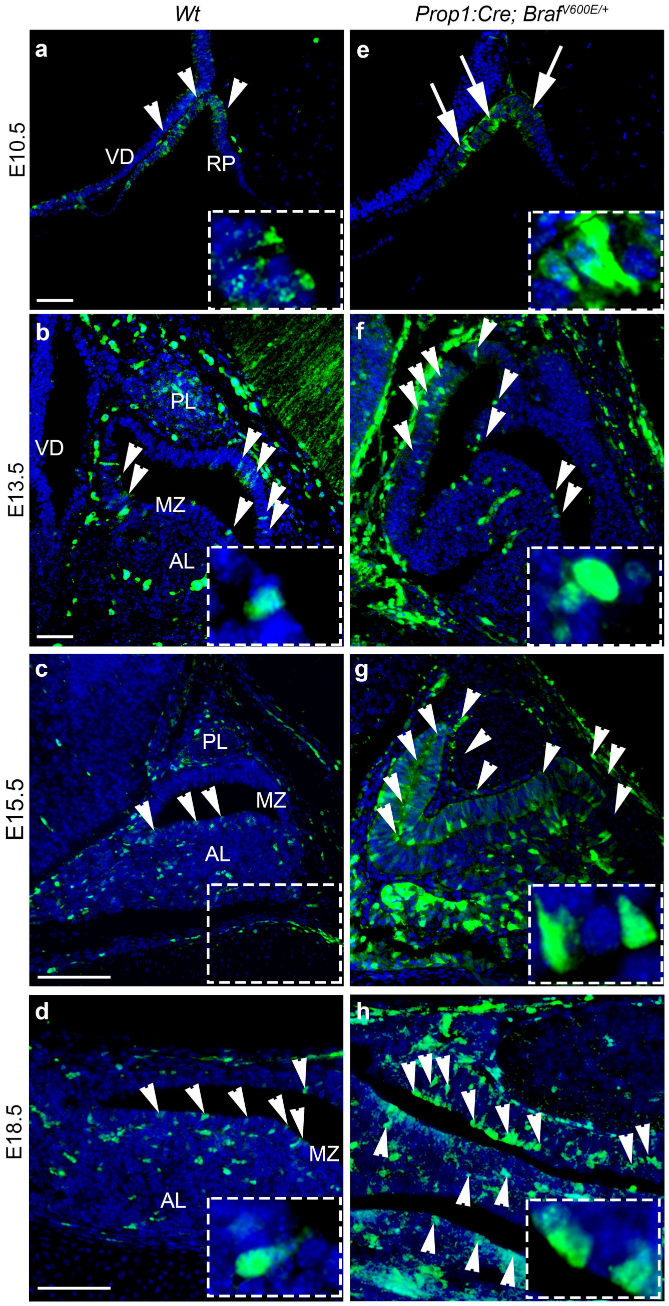

Supplementary Figure 22: Immunofluorescence of pERK reveals increased activated MAPK expression pattern in *Prop1:Cre;Braf*$^{V600E/+}$ embryos. Single immunofluorescence against pERK during pituitary development in Wt (**a**-**d**) and *Prop1:Cre;Braf*$^{V600E/+}$ (**e**-**h**) reveals expression of pERK at E10.5 in the oral ectoderm forming the Rathke's Pouch (RP, arrowheads in **a**) with stronger expression of pERK in the mutant RP (arrows in **e**). Note thickening of the oral ectoderm in the *Prop1:Cre;Braf*$^{V600E/+}$ mutant compared to Wt (arrows **e**). At E13.5 and E15.5, an increased number of pERK+ve cells were observed in mutant pituitaries along the cleft and marginal zone (arrowheads in **f** and **g**) where the PSC reside, whilst only a few sparse pERK+ve cells were observed in the Wt (arrowheads in **b** & **c**). By E18.5, very few pERK+ve cells were observed in the Wt pituitary (arrowheads in **d**), whilst in the mutant pituitary a large number of pERK+ve cells where found lining the marginal zone (arrowheads in **h**). Images are representative of 3 embryos per genotype. Abbreviations: AL, anterior lobe; MZ, marginal zone; PL, posterior lobe; RP, Rathke's Pouch; VD, ventral diencephalon. Scale bars in (**a** & **b**) represent 50 µm and in (**c** & **d**) represent 100 µm.

Supplementary Figure 23

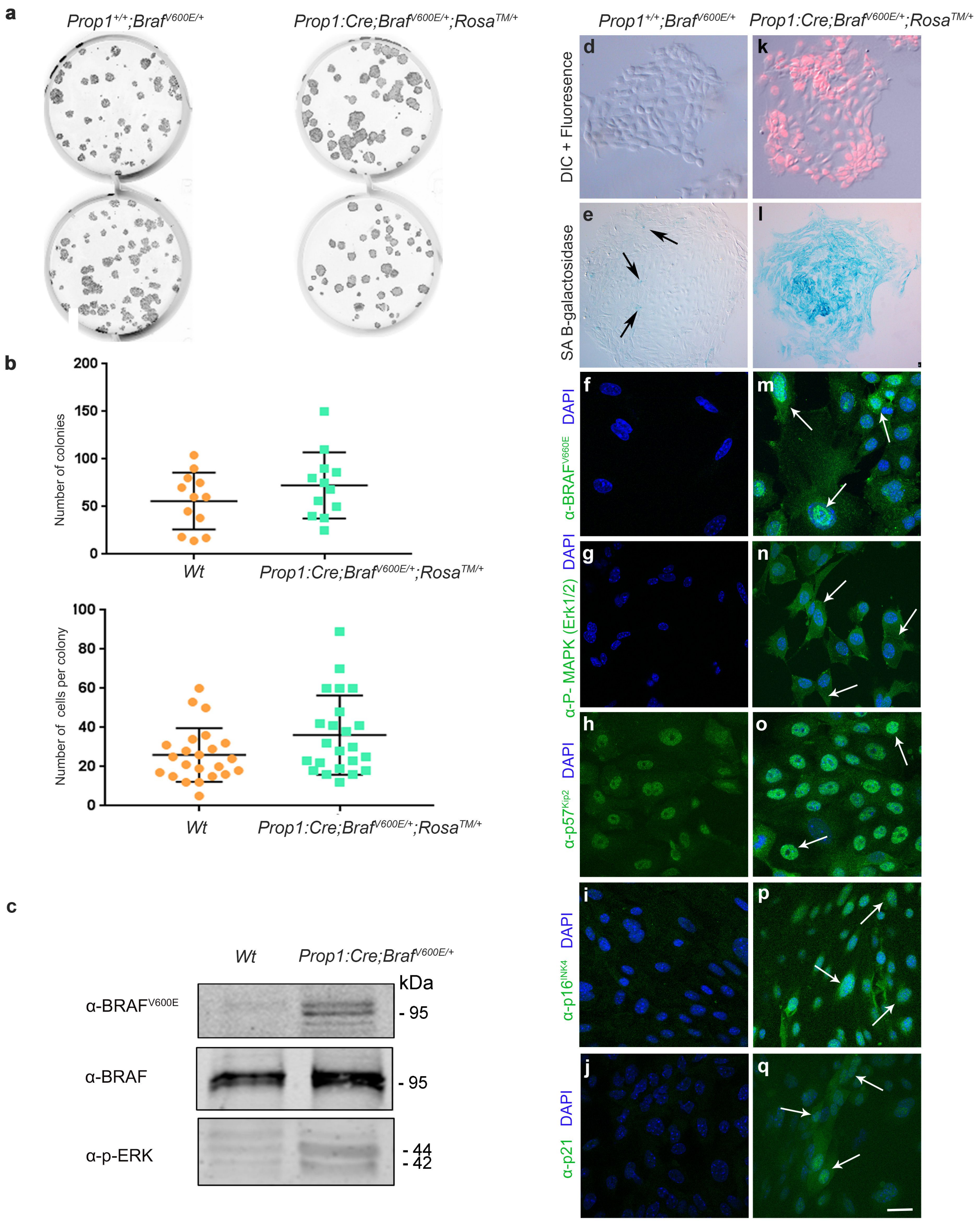

Supplementary Figure 23: Expression of Brafp.V600E in pituitary progenitor/stem cells (PSCs) leads to expression of the senescence markers SA-β-galactosidase, p16^{INK4a}, p21 and cell cycle inhibitors p57^{Kip2}. (a) PSC cultures from Wt or *Prop1:Cre;Braf$^{V600E/+}$;Rosa$^{TM/+}$* at E18.5. **(b)** Quantification revealed no statistical differences in the amount of colonies (p=0.227) or number of cells per colony (p=0.098) indicating that Brafp.V600E does not increase proliferation. P values calculated using an unpaired two-tailed Student's T-test. Data represented as mean ± SEM of n=12 to 23 pituitaries. **(c)** Western blot against Brafp.V600E from PSC lysates from *Prop1:Cre;Braf$^{V600E/+}$* or Wt shows specific expression of Brafp.V600E in mutants (**c**) and increased phosphorylated ERK compared to Wt, indicating activation of the ERK/MAPK pathway. (**d-q**) PSCs from *Prop1:Cre;Braf$^{V600E/+}$;Rosa$^{TM/+}$* mutant pituitaries expressed Tomato florescent protein (**k**) and express Brafp.V600E (arrows in **m**) and cytoplasmic phosphorylated ERK (arrows in **n**) compared to Wt (**f**-**g**, respectively). Mutant PSC colonies express SA-β-galactosidase (**l**) compared to Wt in which only a few cells are SA-β-galactosidase-positive (arrows in **e**). Immunofluorescence against p57^{Kip2} showed upregulation in mutant PSCs (arrows in **l**) compared to Wt (**e**), whilst p16^{INK4a} (arrows in **p**) and p21 (arrows in **q**) were expressed in mutant *Prop1:Cre;Braf$^{V600E/+}$;Rosa$^{TM/+}$* PSC cells and absent in Wt (**h**, **i**, **j**). All images are representative of 3 independent experiments. Scale bar in (**n**) represents 10 µm.

Supplementary Figure 24

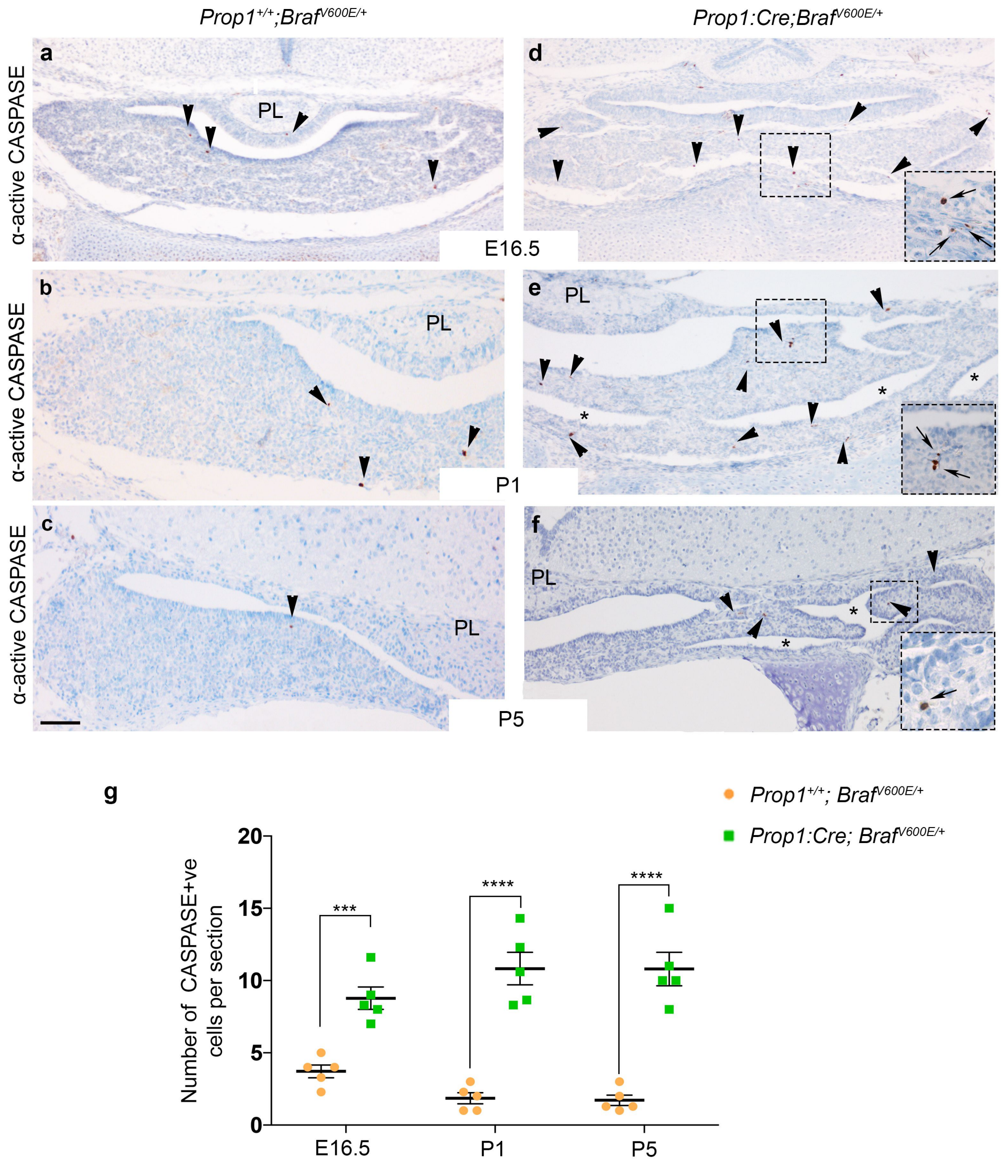

Supplementary Figure 24: Expression of Brafp.V600E results in a significant increase in apoptosis in the *Prop1:Cre;Braf*$^{V600E/+}$ mutant pituitaries. **(a-f)** Coronal sections through the pituitary gland of Wt (**a-c**) and *Prop1:Cre;Braf*$^{V600E/+}$ mutant pituitaries (**d-f**) immunostained against active-CASPASE. Increased number of Caspase positive cells was observed in *Prop1:Cre;Braf*$^{V600E/+}$ mutant pituitaries at E16.5, P1 and P5 (arrowheads in **d-f**) compared to Wt (**a-c**). Insets represent magnified images of the squared areas showing positive foci (arrows in insets). **(g)** Quantification of the number of active-caspase positive cells shows a statistical significant increase in the number of apoptotic cells at E16.5, P1 and P5 (*** p=0.0004 & **** p<0.0001 unpaired two-tailed Student's T-test. Data represented as mean ± SEM of three sections per pituitary of a total of n=5 embryos per genotype). Asterisks in (**e**) and (**f**) denote cavities within the AL. Abbreviations: E, embryonic day; P, postnatal day; PL, posterior lobe. Scale bar in (**c**) represents 100 µm.

Supplementary Figure 25

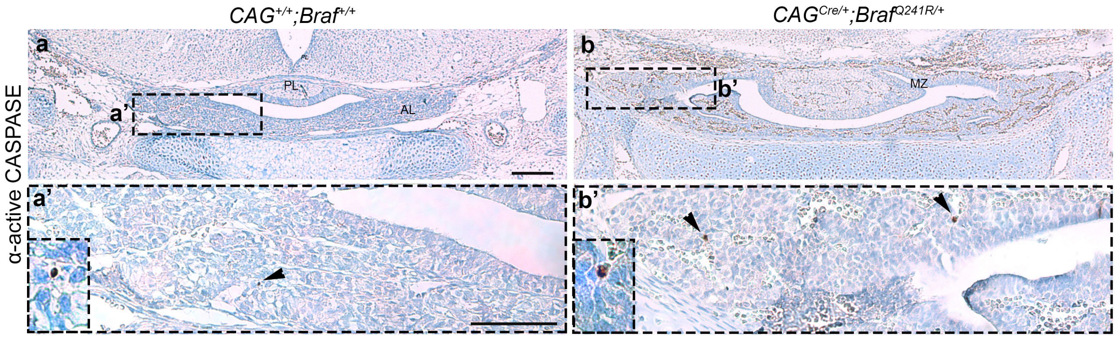

Supplementary Figure 25: The *CAG:Cre;Braf$^{Q241R/+}$* mutant pituitaries have increased apoptosis. **(a, b)** IHC against active-CASPASE in coronal sections through the anterior pituitary gland of E18.5 Wt (**a**) and *CAG:Cre;Braf$^{Q241R/+}$* (**b**) reveals active-CASPASE positive foci in the anterior lobe (arrowheads in **a'** and **b'**). More active-CASPASE positive cells were found in *CAG:Cre;Braf$^{Q241R/+}$* mutant pituitaries compared to Wt. **(a'**-**b')** represent higher magnification images of squared areas in (**a-b**) respectively. Images are representative of 3 independent experiments. Abbreviations: AL, anterior lobe; MZ, marginal zone; PL, posterior lobe. Scale bars in (**a**) and (**a'**) represent 200 μm.

Supplementary Table 1. Endocrine data patient 1-5

	Patient/gender (M/F)	Patient 1 (M)	Patient 2 (F)	Patient 3 (F)	Patient 4 (M)	Patient 5 (M)
BRAF	cDNA reference	c.770 A>G	c.1403T>C	c.721 A>C	c.770 A>G	c.1406G>A
	Protein reference	(p.Q257R)	(p.F468S)	(p.T241P)	(p.Q257R)	(p.G469E)
	Gestation (weeks)	40	36	Term	Term	Term
	Birth weight, kg (SDS)	4.03 (1.02)	2.93 (0.68)	3.74 (0.66)	3.63 (0.14)	N/A
	Current age	16.8y	14.3y	16.4y	17.8y	5.85y
	Height, cm (SDS)	145.3 9 (-4.18)	135 (-4.15)	143.8 (-3.22)	142.5 (-4.8)	100.8 (-2.3)
	Weight, kg (SDS)	34.5 (-4.77)	28.9 (-3.89)	45.4 (-1.55)	38 (-1.05)	20.6 (+0.19)
Endocrine referral	Age at referral	1.9y	0.9y	5.6y	11.1y	3.7y
	Reason for referral	Short stature, hypoglycaemia	MRI features of SOD	Short stature	Short stature	Short stature
	Height SDS	-3.6	-3.1	-3.5	-3.8	-2.15
	BMI SDS	0.3	-4.6	0.9	0.37	2.57
GH axis	Age	2.5	9.7	6.3	12.3	3.9
	Stimulation test	Clonidine	Glucagon	Glucagon	Arginine	Glucagon
	Basal/µg/L	2.0	1.1	5.7	2.0	0.9
	Peak/µg/L (NR)	4.7 (>6.7)	5.1 (>6.7)	11 (>6.7)	4.1 (>6.7)	13.5 (>6.7)
	IGF-1/µg/L (NR)	66 (20-80)	69 (111-551)	74 (88-474)	25.1 (7.8-74)	43 (49-283)
Thyroid axis	**TFTs**					
	fT4 pmol/L, (age/y), NR	16.6 (3.4y), 10.3 (4.1y), 7.5-21.1	9.4 (9.8y), 10.8-19.0	10.8 (6.3y), 7.5-21.1	13.9 (16.0), (9.8-19.2)	15.6 (3.9), 10.8-19
	TSH mU/L, (age/y), NR	0.7 (3.4y), 0.58 (4.1y), 0.34-0.56	3.0 (9.8y), 0.4-4.69.8y3.0, 3.0, 3.0	0.71 (6.3y), 0.34-5.6	1.47 (16.0), (0.5-5)	1.4 (3.9), 0.8-7.2
	**TRH test, age**	4.1y				3.9y
	TSH (0,20,60min) IU/L	0.58, 9.10, 5.30		-	-	1.4, 13, 10.3
Pubertal axis	Tanner stage, age (y)	1, 14.1y	2, 9.7y	3, 15.4y	3, 17.1y	1, 3.9y
	GnRH test, age (y)	14.1y	9.7y	-14.1y	-	3.9y
	LH (0,20,60min) IU/L	1.7, 3.5, 4.1	1.3, 63.7, 64.9	1.0, 55.8, 40.8	0.8, 35.3, 31.0	<0.2, 0.9, 0.8
	FSH (0,20,60min) IU/L	1.1, 4.2, 8.0	7.7, 25.7, 49.7	3.5, 21.2, 21.0	7.7, 15.0, 16.2	0.8, 3.7, 4.8
	Testosterone nmol/L	0.5	-	<18		Basal testosterone <0.69 nmol/L, Peak testosterone 4.51 nmol/L to 3 days of HCG
	Oestradiol pmol/L	-	88			
Adrenal axis	Stimulation test, age (years)	Standard synacthen, 16.0y	Standard synacthen, 9.8y	Modified synacthen, 9.0y	-	Modified synacthen, 3.9y
	Peak cortisol, nmol/L	817	803	433; Standard synacthen at 16.4y, 593		414
	Random cortisols nmol/L (time), age	140, 115, 310 (8am), 7.1y	263 (2pm), 13.6y	N/A	126 (11am), 12.3y	

					673, 18y	
Treatment	Endocrine medication<br><br>(age at start, years)<br><br>Notes	GH<br><br>(3.6-13.4y, restarted 14.4y)<br><br>Trial off caused growth arrest and fall in IGF-1<br><br>Levothyroxine (4.1y)<br><br>Testosterone (14.1y)	GH<br><br>(11.4y)<br>Levothyroxine (4.1y)<br><br>Transdermal estrogen	GH<br><br>(7.7y)<br><br>Estrogen started at 15.4 years There had been pubertal arrest for 12 months prior despite good weight.<br><br>Hydrocortisone (9y), stopped 16.4 years	GH<br><br>(13.0y)	None

Auxological and endocrine data on Patients 1-5. Endocrine values from clinical evaluation and medication administered to each patient are presented here. Abbreviations: *SDS, standard deviation score; : IU, International Units; NR, Normal Range; HCG, Human Chorionic Gonadotrophin; BMI, body mass index; GH, growth hormone; IGF-1, insulin-like growth factor; TFT, thyroid function test; FT4, free thyroxine; TSH, thyroid-stimulating hormone; TRH, thyrotropin-releasing hormone; GnRH, gonadotrophin-releasing hormone; LH, luteinizing hormone; FSH, follicle-stimulating hormone; y, years.

Supplementary Table 2. Clinical features

Patient/gender (M/F)	Patient 1 (M)	Patient 2 (F)	Patient 3 (F)	Patient 4 (M)	Patient 5 (M)
**Dysmorphic facial features**	Macrocephaly, curly hair, wide nasal bridge, down slanting palpebral fissures	Macrocephaly, macroglossia, high anterior hair line, curly hair, posteriorly rotated ears, deep set eyes, wide nasal bridge, everted umbilicus, bilateral single palmar crease	Curly hair, wide spaced eyes, broad nasal tip, depressed nasal bridge, dry skin, multiple extra moles, down-slanting palpebral fissures	Low-set ears, square shaped head, short curly hair, freckles	Macroglossia, umbilical hernia, sensorineural hearing loss, large dysplastic optic nerves
**Gastrointestinal**	Mid-gut malrotation, dysmotility, FTT, GORD, gastrostomy fed, constipation	Poor feeding, gastrostomy fed	Poor feeding	Poor feeding, liquidised food only	Gastro-esophageal reflux with oropharyngeal dysphagia<br>Fundoplication<br>Gastrostomy-fed
**Cardiac**	Mild pulmonary valve stenosis	Pulmonary stenosis – resolved<br>Left ventricular hypertrophy<br>Muscular subaortic valve stenosis	Common AV junction, superior axis deviation	Pulmonary valve stenosis, small secundum ASD, bicuspid/dysplastic aortic valve, mild aortic and mitral valve regurgitation.<br>Intermittent self-limiting ventricular tachycardia	Structurally normal heart
**Neurodevelopment**	Severe global developmental delay, bilateral lower limb hyperreflexia and hypertonia.<br>Vision not impaired	Severe global developmental delay, seizures.<br>Poor vision, bilateral optic nerve hypoplasia	Mild gross motor and speech delay, bilateral squints, poor visual perception, neonatal seizures	Moderate developmental delay, myopia	Global developmental delay
**Renal**	Left renal pelvic dilatation	-	-	Reduced cortico-medullary differentiation	
**Other features**	Capillary haemangioma tip of nose<br>Antenatal polyhydramnios	Antenatal severe polyhydramnios<br>Wheelchair dependent.<br>Scoliosis.<br>Hypotonia.<br>Recurrent respiratory infections<br>Low bone density (lumbar DEXA BMD Z-score -2.9)<br>Hepatic haemangiomas causing hepatomegaly	Mild asthma	Left inguinal hernia (repair age 13y)<br>Meningitis aged 10 years	Antenatal hydramnios<br>Respiratory difficulties, obstructive sleep apnoea, chronic stridor<br>Tracheostomy<br>Hepatosplenomegaly, small retractile right gonad, and a non-palpable left gonad

Supplementary Table 3. Gene panels for hypopituitarism, Septo-Optic dysplasia and cardiofaciocutaneous syndrome

**Disorder**	**Genes**
Congenital Hypopituitarism	*ANOS1, ANXA1, APOE, AR, ARNT2, ASPM, AXL, B9D1, BMP2, BMP4, BMP7, CC2D2A, CCDC141, CCDC88C, CCKBR, CDON, CETP, CGA, CGB1, CGB2, CGB3, CGB7, CHD4, CHD7, CRY1, CTNNB1, CXCR4, CYP19A1, DCC, DCHS1, DHCR7, DUSP6, EBF2, EIF2S3, ESR1, ESR2, F2, F5, FEZF1, FGF13, FGF17, FGF8, FGFR1, FLRT3, FOXA2, FOXL2, FSHB, FSHR, GAP43, GH1, GHR, GLI2, GLI3, GNRH1, GNRHR, GPR161, HESX1, HFE, HHIP, HNRNPU, HS6ST1, ICAM1, IGSF1, IGSF10, IL17RD, INPP5E, JAG1, KCNQ1, KIF14, KISS1, KISS1R, KLB, LHB, LHCGR, LICAM, LHX3, LHX4, MC2R, MC4R, MAGEL2, MTHFR, NDE1, NDN, NFKB2, NOS1, NOTCH1, NR0B1, NR3C1, NR5A1, NRP2, NSMF, NTN1, OTUD4, OTX2, PALM2, PCSK1, PDE3A, PDE5A, PIN1, PNPLA6, POLR3A, POLR3B, POMC, POU1F1, PRLR, PROK2, PROKR2, PROP1, RD3, RELN, RNF216, ROBO1, ROBO2, SELE, SELP, SEMA3A, SEMA3E, SEMA7A, SERPINE1, SHH, SIX6, SLC12A6, SLC15A4, SLC20A1, SLC6A3, SMAD1, SMAD4, SMCHD1, SMPD3, SOX10, SOX2, SOX3, SPRY4, SRA1, STUB1, TAC3, TACR3, TBX19, TCF3, TCF7L1, TF, TGIF1, THRA, THRB, TRAPPC9, TSPAN11, WDR11*
Septo-optic dysplasia	*ANOS1, FGF8, FGFR1, GH1, GHR, HESX1, LHX3, LHX4, OTX2, PAX6, POU1F1, PROKR2, PROP1, RALGAPB, SOX2, SOX3*
Cardiofaciocutaneous syndrome	***BRAF**, HRAS, KRAS, MAP2K1, MAP2K2, NRAS, PPP1R13L, PTPN11, RAF1, RIT1, SHOC2, SOS1*

List of analysed genes for coding and splice region variants in the genes that have been previously associated with congenital hypopituitarism (CH), Septo-optic dysplasia (SOD) and Cardio Facio Cutaneous syndrome (CFC) did not identify any potential pathogenic variants. Only mutations in BRAF.

Supplementary Table 4. Mutagenesis primers

Mutant	Nucleotide Sequence (5'→ 3')
c.1799 T>A (pV600E)	FW: TGGTCTAGCTACAGAGAAATCTCGATGGA<br>RE: TCCATCGAGATTTCTCTGTAGCTAGACCA
c.721 A>C (p.T241P)	FW: ACTTTGTACGAAAACCGTTTTTCACCTTA<br>RE: TAAGGTGAAAAACGGTTTTCGTACAAAGT
c.770 A>G (p.Q257R)	FW: AAAGCTGCTTTTCCGGGGTTTCCGCTGTC<br>RE: GACAGCGGAAACCCCGGAAAAGCAGCTTT
c.1403 T>C (p.F468S)	FW: TGGATCTGGATCATCTGGAACAGTCTACA<br>RE: TGTAGACTGTTCCAGATGATCCAGATCCA
c.1406G>A (p.G469E)	FW: ATCTGGATCATTTGAAACAGTCTACAAGG<br>RE: CCTTGTAGACTGTTTCAAATGATCCAGAT
HindIII<br>Not1	5'-TGCAAGCTTTCTCGGTTATAAGATGGCG<br>5'-CTGGCGGCCGCTTTCAGTGGACAGGAAACG-3'

Oligonucleotide sequence of primers used for site direct mutagenesis to generate hBRAF mutant proteins.

Supplementary Table 5. RT-qPCR primers

Protein (gene)	Nucleotide Sequence (5'→ 3')
P16 ^{INK4a} (*Cdkn2a*)	FW: GTTGTTGAGGCTAGAGAGGATC<br>RE: GTCCTCGCAGTTCGAATCTGCA
P21 (*Cdkn1a*)	FW: GTCGCTGTCTTGCACTCTGG<br>RE: CTCTTGCAGAAGACCAATCTGC
P27^{Kip1} (*Cdkn1b*)	FW: GAAATCTCTTCGGCCCGGTC<br>RE:CACTTGCGCTGACTCGCTTC
P57^{Kip2} (*Cdkn1c*)	FW: AGAGAACTGCGCAAGAGAAC<br>RE: TCTGGCCGTTAGCCTCTAAA

Oligonucleotide sequence of primers used for RT-qPCR.

Supplementary Table 6. **Antibodies**

Antibodies	Source
Rabbit α-Total ERK	1:3000 dilution, Sigma-Aldrich, M5
Mouse α-BRAF	1:3000 dilution, Santa Cruz, sc-5284
Mouse α-β-actin	1:10000 dilution, Santa Cruz Biotechnology
Mouse α-Diphosphorylated ERK	1:1500 dilution, Sigma-Aldrich, M8159
Rabbit α-GAPDH	1:500 dilution, Santa Cruz Biotechnology
Rabbit α-Braf V600E	1:200 dilution, Abcam ab200535
IRDye 800CW Donkey anti-rabbit	1:5000 dilution, LI-COR Biosciences
IRDye 680 anti-mouse antibody	1:5000 dilution, LI-COR Biosciences
Rabbit α-phosphorylated-histone H3	1:300 dilution, MILLIPORE, 06-570
Rabbit α-Caspase	1:200 dilution, Cell Signalling, 9661S
Goat α-td Tomato	1:200 dilution, SICGEN, AB8181-200
Rabbit α-PRL	1:500 dilution, The National Hormone and Peptide Program (NHPP) Harbour-UCLA Medical Centre
Rabbit α-LH	1:500 dilution, The National Hormone and Peptide Program (NHPP) Harbour-UCLA Medical Centre
Rabbit α-TSH	1:500 dilution, The National Hormone and Peptide Program (NHPP) Harbour-UCLA Medical Centre
Rabbit α-GH	1:500 dilution, The National Hormone and Peptide Program (NHPP) Harbour-UCLA Medical Centre
Rabbit α-Pomc	1:500 dilution, The National Hormone and Peptide Program (NHPP) Harbour-UCLA Medical Centre
Rabbit α-GSU	1:500 dilution, The National Hormone and Peptide Program (NHPP) Harbour-UCLA Medical Centre
Rabbit α-TPIT (TBX19)	1:200 dilution, gift from J. Drouin, Montreal Clinical Research Institute
Rabbit α-p57^{Kip2}	1:500 dilution, Abcam, ab4058
Rabbit α-PIT1	1:300 dilution, gift from S. Rhodes, Indiana University School of Medicine, Indianapolis USA
Rabbit α-p16^{INK4a}	1:500 dilution, Abcam, ab51243
Mouse α-p27^{Kip1}	1:600 dilution, Santa Cruz, sc1641
Mouse α-p21	1:500 dilution, Santa Cruz, F0817
Rabbit α-p57 ^{Kip2}	1:200 dilution, Abcam, ab75974
Mouse α- p27 ^{Kip1}	1:200 dilution, BD, 610242
Goat α- Sox2	1:200 dilution, Neuromics, GT15098
Rat α- BrdU	1:500 dilution, Abcam, Ab6326
Rabbit α- Phospho-p44/42 MAPK (Erk1/2) (Thr202/Tyr204)	1:100 dilution, Cell Signalling, 9101
Alexa Fluor™ 488 Tyramide SuperBoost™ Kit, streptavidin	Thermo Fisher, B40932
ApopTag® Plus In Situ Apoptosis Fluorescein Detection Kit	Merck, S7111
biotinylated goat anti-rabbit antibody	1:300 dilution, Vector Laboratories, BA-1000
biotinylated horse anti-goat antibody	1:200 dilution, Vector Laboratories, BA-9500
donkey anti-goat antibody, Alexa Fluor 568	1:300, Invitrogen, A-11057
goat anti-mouse antibody, Alexa Fluor 488	1:300, Invitrogen, A-11001

Supplementary Table 7. **Genotyping primers**

Gene	Nucleotide Sequence (5' →3')
Braf:V600E	FW: TGAGTATTTTTGTGGCAACTGC<br>RE: CTCTGCTGGGAAAGCGGC
Prop:Cre	FW: TCACTACCGGGCGTATTTTT<br>RE: GCCGGATAAAACTTGTGCTT
Rosa:Tm mutant allele<br><br>*Rosa*:Tm wild type allele	FW: GGCATTAAAGCAGCGTATCC<br>RE: CTGTTCCTGTACGGCATGG<br><br>FW: AAGGGAGCTGCAGTGGAGTA<br>RE: CCGAAAATCTGTGGGAAGTC

Oligonucleotide sequence of primers used for genotping.

Uncropped western blot gels used for Figure 3 C

A

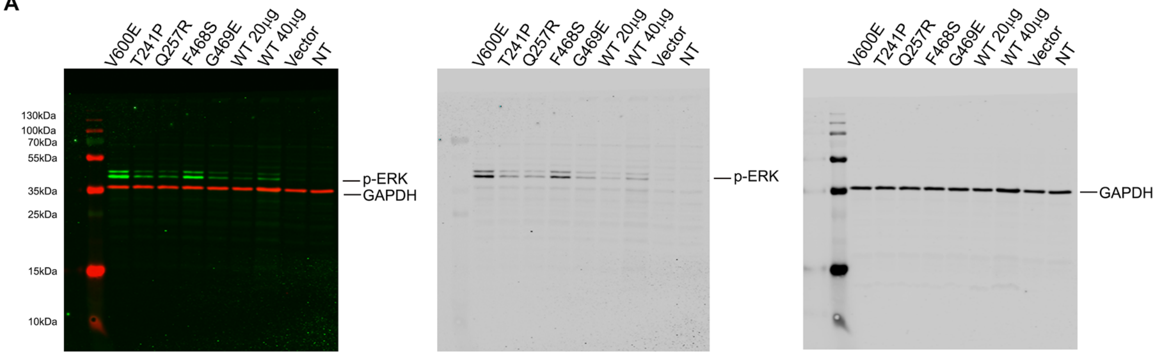

B

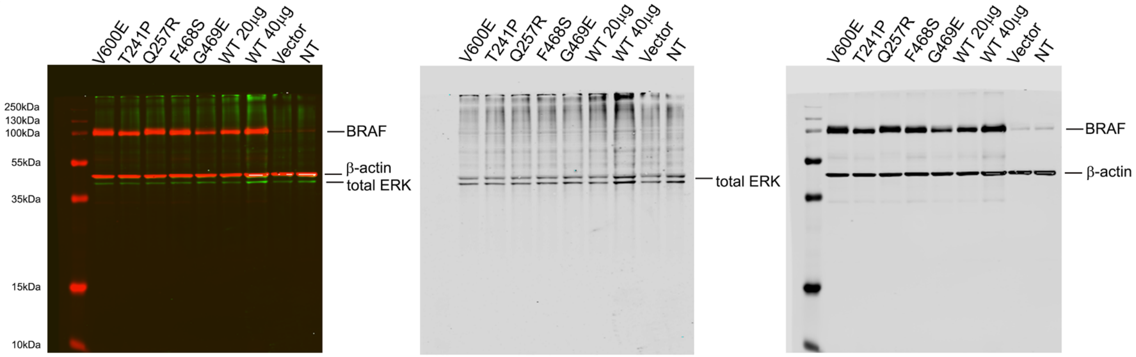

Figure 10. (m) uncropped gels

Gel1

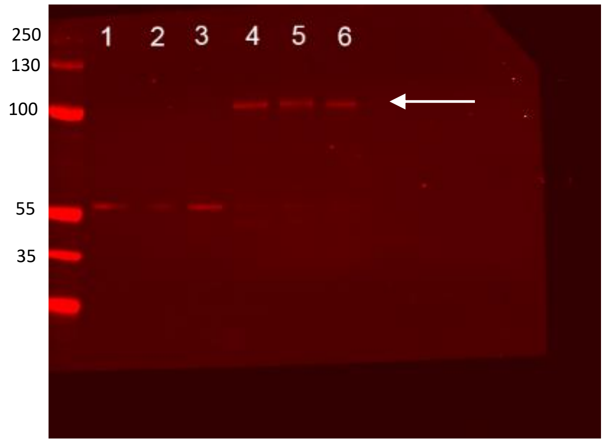

Lysates lanes 1, 2, 3 Wt
Lysates lanes 3, 4, 5 Mutant

Lane 3&4 used in Figure 10.M

α-BRAFV600E

Gel2

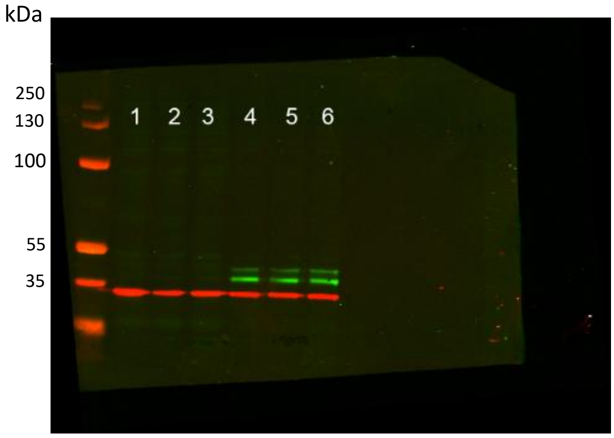

Lysates lanes 1, 2, 3 Wt
Lysates lanes 3, 4, 5 Mutant

Lane 3&4 used in Figure 10.M

Green α-pERK
Red α-GAPDH

Uncropped WB Figure 23 C showing expression of BRAFp.V600E

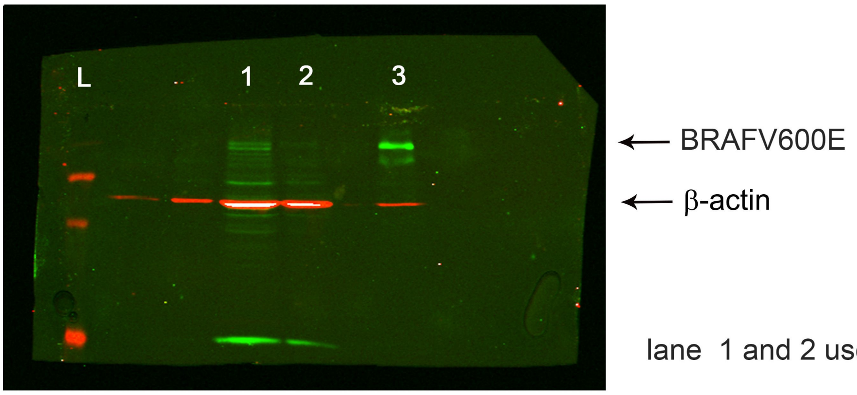

lane 1 and 2 used in Figue 10C

L Ladder
1 Mutant Lysate: from *Prop1Cre;Braf*$^{V600E/+}$*;Rosa*$^{TM/+}$
2 Wild type Lysate from *Prop1*$^{+/+}$*,Braf*$^{+/+}$
3 Control lysate from 293HEK cells trasnfected with human BRAFV600E

Uncropped WB Figure 23 C showing expression total BRAF

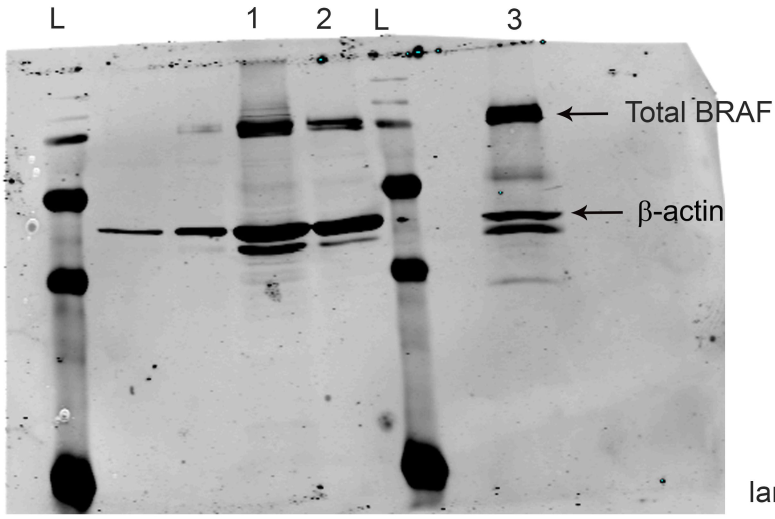

lane 1 and 2 used in Figue 10C

L Ladder
1 Mutant Lysate: from *Prop1Cre;Braf*$^{V600E/+}$*;Rosa*$^{TM/+}$
2 Wild type Lysate rom *Prop1*$^{+/+}$*,Braf*$^{+/+}$
3 Control lysate 293HEK cells trasnfected with human BRAFV600E

Uncropped WB Figure 23 C showing expression of phosphorylated-ERK

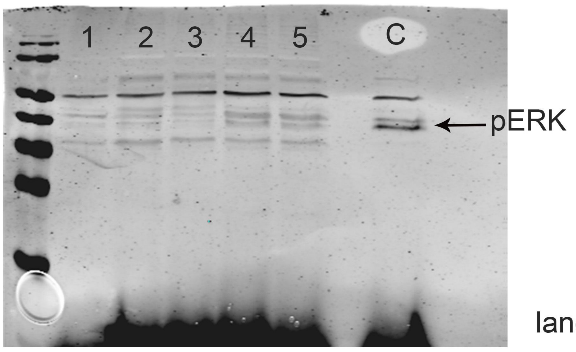

lane 3 and 4 used in Figue 10C

1,2,3 Wt lysate
4,5 Mutant Lysate: from *Prop1Cre;Braf*$^{V600E/+}$*;Rosa*$^{TM/+}$
C Control Laysate 293HEK cells transfected with BRAFV600E
