# Supplementary material for: Activating mutations in BRAF disrupt the hypothalamo-pituitary axis leading to hypopituitarism in mice and humans
Source: Nat Commun. 2021 Apr 1;12:2028. doi: 10.1038/s41467-021-21712-4 (PMC8016902; doi:10.1038/s41467-021-21712-4)
Supplement: Supplementary file 3 — Reporting Summary [file 41467_2021_21712_MOESM3_ESM.pdf]

nature research

Corresponding author(s): Carles Gaston-Massuet

Last updated by author(s): 19/03/2020

Reporting Summary

Nature Research wishes to improve the reproducibility of the work that we publish. This form provides structure for consistency and transparency in reporting. For further information on Nature Research policies, see Authors & Referees and the Editorial Policy Checklist .

Statistics

For all statistical analyses, confirm that the following items are present in the figure legend, table legend, main text, or Methods section.

n/a	Confirmed	
☐	☒	The exact sample size (*n*) for each experimental group/condition, given as a discrete number and unit of measurement
☐	☒	A statement on whether measurements were taken from distinct samples or whether the same sample was measured repeatedly
☐	☒	The statistical test(s) used AND whether they are one- or two-sided<br>*Only common tests should be described solely by name; describe more complex techniques in the Methods section.*
☐	☒	A description of all covariates tested
☒	☐	A description of any assumptions or corrections, such as tests of normality and adjustment for multiple comparisons
☐	☒	A full description of the statistical parameters including central tendency (e.g. means) or other basic estimates (e.g. regression coefficient) AND variation (e.g. standard deviation) or associated estimates of uncertainty (e.g. confidence intervals)
☐	☒	For null hypothesis testing, the test statistic (e.g. *F*, *t*, *r*) with confidence intervals, effect sizes, degrees of freedom and *P* value noted<br>*Give P values as exact values whenever suitable.*
☒	☐	For Bayesian analysis, information on the choice of priors and Markov chain Monte Carlo settings
☒	☐	For hierarchical and complex designs, identification of the appropriate level for tests and full reporting of outcomes
☐	☒	Estimates of effect sizes (e.g. Cohen's *d*, Pearson's *r*), indicating how they were calculated

Our web collection on statistics for biologists contains articles on many of the points above.

Software and code

Policy information about availability of computer code

Data collection

Exome Sequencing:

Whole exome capture and sequencing was performed at BGI (Shenzhen, China) using SureSelect Human All Exon v6 60 Mb kit (Agilent Technologies, Santa Clara, CA, USA) and BGISEQ-500 platform (Illumina, San Diego, CA, USA). Sequencing reads were aligned with Burrows-Wheeler Aligner (BWA) v0.7.17 79 to human genome build 38 (GRCh38.p1) not including alternate assemblies (GCA_000001405.15_GRCh38_no_alt_analysis_set.fna). Read duplicates were marked with Sambamba 80 version 0.6.6.

Phosphoproteomic analyses:

Phosphopeptide pellets were re-suspended in reconstitution buffer (20 fmol/µl enolase in 3% ACN, 0.1% TFA) and loaded onto an LC-MS/MS system consisting of an Ultimate nanoflow ultrahigh pressure liquid chromatograph connected to an Orbitrap Q-Exactive Plus mass spectrometer (Thermo Fisher Scientific) operated with a parameter setting previously described. Spectra were acquired and analysed using XCalibur (Thermo Scientific v4.2 SP1). Peptide identification from MS data was automated with Mascot Daemon2.5.0. Searches were performed against the SwissProt Database (uniprot_sprot_2014_08.fasta) using the Mascot search engine (v2.5) and the parameters described in. The in house developed software Pescal was used for label-free peptide quantification, Pescal constructed extracted ion chromatograms (XIC) for each identified peptide and measured the area of the XICs for all the peptides identified across all samples. Thresholds for XIC generation were ±7 ppm and ±2 min m/z and retention time windows, respectively, undetectable peptides were assigned a value equal to the lowest detected intensity across sample divided by 10. Pescal (v01) outputs were exported to XLS files and further processed with Microsoft Excel 2016. Values of 2 technical replicates per sample were averaged and intensity values for each peptide were normalized to total sample intensity. Differences in peptide phosphorylation between Wt and BRAF variants were reported as fold over Wt and statistical significance for those changes was assessed using unpaired two tailed t-test. Kinase activities from phosphoproteomics data were inferred by KSEA as described before. The calculation of KSEA values for each kinase was automated using a VBA script written in house.

Data analysis	Statistical analyses were performed using Graph-PAD-Prism Version9 and cell counts using ImageJ

For manuscripts utilizing custom algorithms or software that are central to the research but not yet described in published literature, software must be made available to editors/reviewers. We strongly encourage code deposition in a community repository (e.g. GitHub). See the Nature Research guidelines for submitting code & software for further information.

Data

Policy information about availability of data

All manuscripts must include a data availability statement. This statement should provide the following information, where applicable:
- Accession codes, unique identifiers, or web links for publicly available datasets
- A list of figures that have associated raw data
- A description of any restrictions on data availability

All mass spectrometry proteomics data have been deposited to the ProteomeXchange Consortium via the PRIDE partner repository with the dataset identifier PXD018190.

Field-specific reporting

Please select the one below that is the best fit for your research. If you are not sure, read the appropriate sections before making your selection.

[x] Life sciences [ ] Behavioural & social sciences [ ] Ecological, evolutionary & environmental sciences

For a reference copy of the document with all sections, see nature.com/documents/nr-reporting-summary-flat.pdf

Life sciences study design

All studies must disclose on these points even when the disclosure is negative.

Sample size	Sample sizes were inferred by analyses of two mean (Student's T- test) or more than two independent groups (ANOVA) to have a power of 0.95 with a p<0.05. This was based on either preliminary pilot studies or already published data.
Data exclusions	No data was excluded in our studies
Replication	Experiments are representative of a minimum three independent experiments that contain 3 experimental replicates each. In other instances up to 9 independent experiments were performed. All attempts at replication were successful. Wherever possible blinded experiments for genotypes were conducted. In some instances this was not possible due to the obvious morphological abnormalities seen in the pituitary gland which correlates to the mutant phenotype. Intra and inter-litter variations were taken into account, and embryos were taken from different pregnancies.
Randomization	In our murine and pituitary stem cells experiments groups were separated by their respective genotypes, and embryos compared between several separate litters from different breeding pairs. Animals from different cages within experimental groups were selected. For the validation of the functional role of the BRAF mutations, each individual genetic variant was categorized individually, no randomization was required for this experiment.
Blinding	Whenever possible, blinding of the murine genotypes was conducted to avoid bias. This was not possible when the phenotype was clearly present and visible i.e dwarfism, or severe hypoplasia of pituitary gland in the mutants . Experiments of the functional effect of BRAF mutations and the in vitro colony formation cell culture of pituitary stem cells were all performed blinded to the researcher.

Reporting for specific materials, systems and methods

We require information from authors about some types of materials, experimental systems and methods used in many studies. Here, indicate whether each material, system or method listed is relevant to your study. If you are not sure if a list item applies to your research, read the appropriate section before selecting a response.

Materials & experimental systems

n/a	Involved in the study	
[ ]	[x]	Antibodies
[ ]	[x]	Eukaryotic cell lines
[x]	[ ]	Palaeontology
[ ]	[x]	Animals and other organisms
[ ]	[x]	Human research participants
[x]	[ ]	Clinical data

Methods

n/a	Involved in the study	
[x]	[ ]	ChIP-seq
[x]	[ ]	Flow cytometry
[ ]	[x]	MRI-based neuroimaging

Antibodies

Antibodies used	Antibodies used are described in supplementary materials (Supplementary Table 6) with the source and concentration used:<br><br>Rabbit α-Total ERK (1:3000 dilution, Sigma-Aldrich, M5); Mouse α-BRAF (1:3000 dilution, Santa Cruz, sc-5284); Mouse α-β-actin (1:3000 dilution, Santa Cruz, sc-5284); Mouse α-Diphosphorylated ERK (1:1500 dilution, Sigma-Aldrich, M8159); Rabbit α-GAPDH (1:500 dilution, Santa Cruz Biotechnology) ; Rabbit α-Braf V600E (1:200 dilution, Abcam ab200535); IRDye 800CW Donkey anti-rabbit (1:5000 dilution, LI-COR Biosciences); IRDye 680 anti-mouse antibody (1:5000 dilution, LI-COR Biosciences); Rabbit α-phosphorylated-histone H3 (1:300 dilution, MILLIPORE, 06-570); Rabbit α-Caspase (1:200 dilution, Cell Signalling, 9661S); Goat α-td Tomato (1:200 dilution, SICGEN, AB8181-200); Rabbit α-PRL, Rabbit α-LH, Rabbit α-TSH, Rabbit α-GH, Rabbit α-Pomc, Rabbit α-GSU (1:500 dilution, The National Hormone and Peptide Program (NHPP) Harbour-UCLA Medical Centre); Rabbit α-TPIT (TBX19) (1:200 dilution, gift from J. Drouin, Montreal Clinical Research Institute); Rabbit α-p57Kip2 (1:500 dilution, Abcam, ab4058); Rabbit α-PIT1 (1:300 dilution, gift from S. Rhodes, Indiana University School of Medicine, Indianapolis USA); Rabbit α-p16INK4a (1:500 dilution, Abcam, ab51243); Mouse α-p27Kip1 (1:600 dilution, Santa Cruz, sc1641); Mouse α-p21 (1:500 dilution, Santa Cruz, F0817); Rabbit αp57 Kip2 (1:200 dilution, Abcam, ab75974); Goat α- Sox2 (1:200 dilution, Neuromics, GT15098); Rat α- BrdU (1:500 dilution, Abcam, Ab6326); Rabbit α- Phospho-p44/42 MAPK (Erk1/2) (Thr202/ Tyr204) 1:100 dilution, Cell Signalling, 9101; Alexa Fluor™ 488 Tyramide SuperBoost™ Kit, streptavidin (Thermo Fisher, B40932); ApopTag® Plus In Situ Apoptosis Fluorescein Detection Kit (Merck, S7111); biotinylated goat anti-rabbit antibody 1:300 dilution, Vector Laboratories, BA-1000); biotinylated horse anti-goat antibody (1:200 dilution, Vector Laboratories, BA-9500); donkey anti-goat antibody, Alexa Fluor 568 (1:300, Invitrogen, A-11057); goat anti-mouse antibody, Alexa Fluor 488 (1:300, Invitrogen, A-11001).
Validation	All antibodies were published and validated in previous studies. All commercial antibodies were validated for the species (mouse) and application (immunostaining) by the correspondent manufacturer, which is described in the manufacturer's website. General protocol is explained in detail in methods sections of the manuscript.

Eukaryotic cell lines

Policy information about cell lines

Cell line source(s)	293Hek Cells lines were commercially obtained (Thermo Fisher Scientific)
Authentication	The cell line used was not authenticated.
Mycoplasma contamination	Mycoplasma test was performed every week on cells using PCR and they tested negative.
Commonly misidentified lines (See ICLAC register)	no commonly misidentified cell lines were used in the study

Animals and other organisms

Policy information about studies involving animals; ARRIVE guidelines recommended for reporting animal research

Laboratory animals	Mouse (mus musculus). The transgenic lines Rosa26CAGLoxpSTOPLoxpTdTomato (stock #007905), BrafV600E/+ (stock #017837) were obtained from the JAX lab and have been previously described. The Prop1:Cre transgenic line was kindly provided by Shannon Davis and Sally Camper. The CAG:Cre;BrafQ241R/+ mice were provided by Shin-ichi Inoue and Yoko Aoki.<br><br>Mice were kept in a 12h dark/light cycle, with constant supply of food and water, temperatures of 65-75°F (~18-23°C) with 40-60% humidity.
Wild animals	none
Field-collected samples	none
Ethics oversight	All experiments were conducted under the regulations, licenses and local ethical review of the UK Home Office (in accordance with the UK law, Animals Scientific Procedures Act 1986) all protocols were approved by the Home Office under the PPL 70/8269.

Note that full information on the approval of the study protocol must also be provided in the manuscript.

Human research participants

Policy information about studies involving human research participants

Population characteristics	Patients with CFC were recruited to the study, and Sanger sequencing performed in regional accredited Genetics laboratories. Ethical committee approval was obtained from the UCL Great Ormond Street Hospital for Children Joint Research Ethics Committee (09/H0706/66). Informed written consent was obtained from all patients and/or parents. Patients presented with the rare disease CFC and congenital hypopituitarism.

Recruitment	This is a rare genetic condition and there was therefore no self-selection bias. Patients were recruited from clinics as they presented with relevant clinical features, and sequencing of BRAF was performed, followed by WES to ensure that there were no other likely causative variants. Patients with CFC and congenital hypopituitarism. No self-selection bias of patients with CFC. 5 patients diagnosed with CFC presented with clinical features of congenital hypopituitarism. The age of the patients ranged from 0.9-11.1 years. The gender ratio was 3 females to 2 males. This is a rare condition and there was therefore no self-selection bias. Patients were recruited from clinics as they presented with relevant clinical features, and sequencing of BRAF was performed, followed by WES to ensure that there were no other likely causative variants.
Ethics oversight	Patients with CFC were recruited to the study, and Sanger sequencing performed in regional accredited Genetics laboratories . Ethical committee approval was obtained from the UCL Great Ormond Street Hospital for Children Joint Research Ethics Committee (09/H0706/66). Informed written consent was obtained from all patients and/or parents to publish clinical data

Note that full information on the approval of the study protocol must also be provided in the manuscript.

Magnetic resonance imaging

Experimental design

Design type	MRI images were only taken to observed morphology of anterior pituitary gland, and features Septo Optic Dysplasia
Design specifications	No experimental design was required in our study as MRI images are only diagnostic
Behavioral performance measures	None (not applicable to our study)

Acquisition

Imaging type(s)	MRI images were acquired on a 1.5 Tesla MRI scanner. Standard T1 weighted sequence of the whole brain were performed at 3mm slice thickness in the sagittal and coronal planes.
Field strength	Field Strength 1.5Tesla
Sequence & imaging parameters	Sequence and imaging parameters 3 mm slices
Area of acquisition	*State whether a whole brain scan was used OR define the area of acquisition, describing how the region was determined.*

Diffusion MRI ☐ Used ☒ Not used

Preprocessing

Preprocessing software	NA
Normalization	NA
Normalization template	NA
Noise and artifact removal	NA
Volume censoring	NA

Statistical modeling & inference

Model type and settings	Not applicable to our study as we did not generate quantifiable data from the MRI. MRIs were used only for description diagnosis.
Effect(s) tested	*Define precise effect in terms of the task or stimulus conditions instead of psychological concepts and indicate whether ANOVA or factorial designs were used.*

Specify type of analysis: ☐ Whole brain ☐ ROI-based ☐ Both

Statistic type for inference (See Eklund et al. 2016)	*Specify voxel-wise or cluster-wise and report all relevant parameters for cluster-wise methods.*
Correction	*Describe the type of correction and how it is obtained for multiple comparisons (e.g. FWE, FDR, permutation or Monte Carlo).*

Models & analysis

n/a	Involved in the study
☒	☐ Functional and/or effective connectivity
☒	☐ Graph analysis
☒	☐ Multivariate modeling or predictive analysis
